# Depression and anxiety in patients with active ulcerative colitis: crosstalk of gut microbiota, metabolomics and proteomics

**DOI:** 10.1080/19490976.2021.1987779

**Published:** 2021-11-21

**Authors:** Xiaomin Yuan, Biqing Chen, Zhenglan Duan, Ziqian Xia, Yang Ding, Tuo Chen, Huize Liu, Baosheng Wang, Bolin Yang, Xiaoyong Wang, Shijia Liu, Jin-Yong Zhou, Yajun Liu, Qiong Wang, Zhaofeng Shen, Jun Xiao, Hongtao Shang, Weiwei Liu, Guoping Shi, Lei Zhu, Yugen Chen

**Affiliations:** a Department of Colon and Rectum Surgery, Jiangsu Province Hospital of Chinese Medicine, Affiliated Hospital of Nanjing University of Chinese Medicine, Nanjing, Jiangsu, P. R. China; b Central Laboratory/Research Center of Chinese Medicine, Jiangsu Province Hospital of Chinese Medicine, Affiliated Hospital of Nanjing University of Chinese Medicine, Nanjing, Jiangsu, P.R.China; c Centre of Brain Disease, Jiangsu Province Hospital of Chinese Medicine, Affiliated Hospital of Nanjing University of Chinese Medicine, Nanjing, Jiangsu, P.R.China; d Department of Pharmacy, Jiangsu Province Hospital of Chinese Medicine, Affiliated Hospital of Nanjing University of Chinese Medicine, Nanjing, Jiangsu, P.R.China; e Department of Gastroenterology, Jiangsu Province Hospital of Chinese Medicine, Affiliated Hospital of Nanjing University of Chinese Medicine, Nanjing, Jiangsu, P.R.China; f Laboratory of Pharmacology, Jiangsu Province Hospital of Chinese Medicine, Affiliated Hospital of Nanjing University of Chinese Medicine, Nanjing, Jiangsu, P.R.China; g Department of Science and Technology, Jiangsu Province Hospital of Chinese Medicine, Affiliated Hospital of Nanjing University of Chinese Medicine, Nanjing, Jiangsu, P.R.China; h Gastrointestinal Endoscopy Center, Jiangsu Province Hospital of Chinese Medicine, Affiliated Hospital of Nanjing University of Chinese Medicine, Nanjing, Jiangsu, P.R.China; i Medical Examination Center, Jiangsu Province Hospital of Chinese Medicine, Affiliated Hospital of Nanjing University of Chinese Medicine, Nanjing,Jiangsu, P.R.China; j Collaborative Innovation Center for Cancer Medicine, Jiangsu Province Hospital of Chinese Medicine, Affiliated Hospital of Nanjing University of Chinese Medicine, Nanjing, Jiangsu, P.R.China

**Keywords:** Ulcerative colitis, microbiota-gut-brain axis, multi-omics analyses

## Abstract

Patients with ulcerative colitis (UC) have a high prevalence of mental disorders, such as depression and anxiety. Gut microbiota imbalance and disturbed metabolism have been suggested to play an important role in either UC or mental disorders. However, little is known about their detailed multi-omics characteristics in patients with UC and depression/anxiety. In this prospective observational study, 240 Chinese patients were enrolled, including 129 patients with active UC (69 in Phase 1 and 60 in Phase 2; divided into depression/non-depression or anxiety/non-anxiety groups), 49 patients with depression and anxiety (non-UC), and 62 healthy people. The gut microbiota of all subjects was analyzed using 16S rRNA sequencing. The serum metabolome and proteome of patients with UC in Phase 2 were analyzed using liquid chromatography/mass spectrometry. Associations between multi-omics were evaluated by correlation analysis. The prophylactic effect of candidate metabolites on the depressive-like behavior of mice with colitis was investigated. In total, 58% of patients with active UC had depression, while 50% had anxiety. Compared to patients with UC without depression/anxiety, patients with UC and depression/anxiety had lower fecal microbial community richness and diversity, with more *Lactobacillales, Sellimonas, Streptococcus*, and *Enterococcus* but less *Prevotella_9* and *Lachnospira*. Most metabolites (e.g., glycochenodeoxycholate) were increased in the serum, while few metabolites, including 2ʹ-deoxy-D-ribose and L-pipecolic acid, were decreased, accompanied by a general reduction in immunoglobulin proteins. These related bacteria, metabolites, and proteins were highly connected. A prophylactic administration of 2ʹ-deoxy-D-ribose and L-pipecolic acid significantly reduced the depressive-like behaviors in mice with colitis and alleviated the inflammatory cytokine levels in their colon, blood and brain. This study has identified a comprehensive multi-omics network related to depression and anxiety in active UC. It is composed of a certain set of gut microbiota, metabolites, and proteins, which are potential targets for clinical intervention for patients with UC and depression/anxiety.

## 1. Introduction

Ulcerative colitis (UC) is a chronic inflammatory bowel disease (IBD) that affects the colon and rectum, and its incidence is rapidly increasing in developing countries.^1^

Patients with UC usually experience mental symptoms, such as depression and anxiety, which might be due to the chronicity of UC and impairment of daily life.^2–4^ As reported in a recent meta-analysis, the prevalence of depression and anxiety was 23% and 32.6% in patients with UC, respectively. Moreover, 41.3% of patients with active UC had depression, while 70.8% had anxiety.^5^ On the other hand, patients with a history of depression are more likely to develop UC.^6^ The disease activity of UC was correlated with the severity of depression and anxiety.^7–9^ The treatment designated for one could be effective for the other.^10,11^ However, clinicians have not paid enough attention to the assessment and treatment of depression and anxiety in patients with UC. About one third of patients with UC and depression and two thirds of patients with UC and anxiety have not been diagnosed, and most patients with UC have not received an adequate and efficient psychological treatment in time.^3^ Therefore, a comprehensive understanding of the mechanism of UC-related depression and anxiety is of great clinical significance for the treatment of UC-related comorbidities and for the improvement of the quality of life of patients with UC.

There is a mutual influence between depression/anxiety and UC.^12–14^ The gut-brain bidirectional communication has been indicated as a possible basis for their association.^15^ On one hand, UC could be potentially caused by a disturbed inflammatory response toward a set of specific gut microbiota in genetically susceptible individuals. Gut microorganisms act on the host’s innate immune cells; then, these activate inflammasomes and a series of immune cells (such as T helper cell, regulatory T-cell, and B-cell), to initiate either a pro- or anti-inflammatory response.^16^

On the other hand, the gut microbiota interacts with the central nervous system through the immune system (mainly microglia in the brain) and circulating metabolites (such as neurotransmitters).^17^ The gut microbiota can produce neurotransmitters, such as dopamine, serotonin, and γ-aminobutyric acid (GABA), which can affect the mood.^18–20^

**Figure 1.**
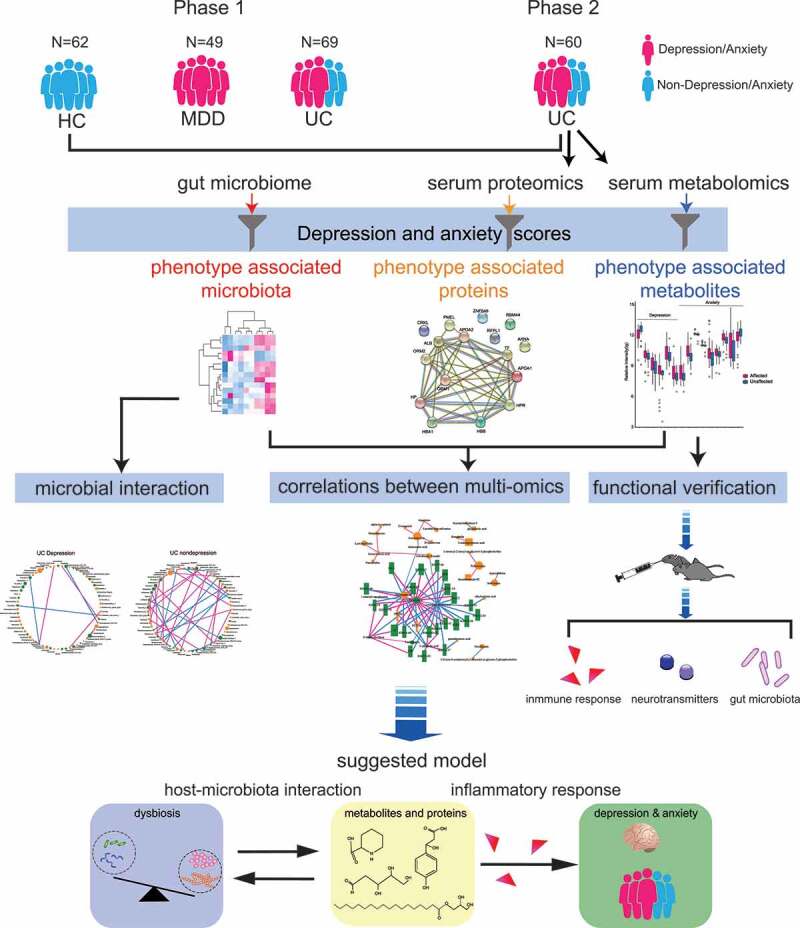
Overview of the workflow. UC, patients with ulcerative colitis; HC, healthy control; MDD, patients without IBD but with depression and anxiety

In recent years, research using multi-omics approach has been widely applied in mechanistic studies on a single disease or on the relationship between two diseases.^21–23^ Therefore, in this study, a multi-omics approach that integrates the gut microbiota, serum metabolome, and serum proteome was adopted to characterize UC-related depression and anxiety (Figure 1). The specific composition of the gut microbiota was found to be associated with depression and anxiety in patients with active UC. Serum metabolome and proteome were profiled to explore potential pathways between the gut microbiota and the brain. The beneficial effects of candidate metabolites and their influence on inflammation were tested on mice with colitis and depressive/anxious-like behaviors.

## 2. Materials and methods

### 2.1. Study cohorts

Participants (n = 240) aged 18–65 years were recruited from the Jiangsu Province Hospital of Chinese Medicine. This study was approved by the Institutional Review Board of the Affiliated Hospital of Nanjing University of Chinese Medicine, with written informed consents obtained from all participants. This is a single-center, prospective, and observational cross-sectional study consisting of three groups in two phases: 129 patients with active UC recruited in two phases (69 in Phase 1 and 60 in Phase 2), 49 patients with depression and anxiety but without IBD(MDD), and 62 healthy subjects (health control, HC). Patient Health Questionnaire-9 (PHQ-9) and Generalized Anxiety Disorder Scale (GAD-7) were used to evaluate their depression and anxiety levels, respectively. PHQ-9 and GAD-7 scores over four points were defined as depression and anxiety, respectively.^24,25^ Patients presented at the outpatient department of psychology were screened using an IBD-related questionnaire (Supplementary Material), PHQ-9, and GAD-7, and those who had both PHQ-9 and GAD-7 scores over four points with no IBD symptoms were recruited into the MDD group. According to the American College of Gastroenterology clinical guideline for UC, a widely recognized standard, the Mayo score, was chosen for the evaluation of UC disease activity.^26^ It is composed of four clinical and endoscopic subscores that ranges from 0 to 12 (none to most severe). Active UC was defined as a Mayo score over two points and an endoscopic subscore over zero. Depression and anxiety levels were assessed on the day of Mayo score evaluation, and all samples were collected on the same day. Any subjects with self-reported mental health problems other than depression or anxiety were excluded from subsequent analyses. Healthy subjects matched in age and sex without IBD or any mental disorders were recruited from the medical examination center, and only those with both PHQ-9 and GAD-7 scores less than five were included.

### 2.2. Microbial profiling

Fecal samples of all participants were collected and prepared, as described in a supplementary method.^27^ DNA was extracted from the stool samples, and the V3–V4 hypervariable region of bacterial 16S rRNA gene was sequenced. Sequences were clustered into OTUs at a similarity level of 97%. OTUs with relative abundances above 0.5% of the total sequences in at least one sample were kept. PICRUSt version 1.1.4 was used to predict enriched pathways.^28^

### 2.3. Metabolomics profiling

Fasting serum samples of each patient with UC at Phase 2 were collected and preprocessed. Ultra-high-performance liquid chromatography-quadrupole time-of-flight tandem mass spectrometry was performed on an Agilent 1290 Infinity LC system (Agilent Technologies, Santa-Clara, California, USA) coupled with an AB SCIEX Triple TOF 6600 System (AB SCIEX, Framingham, MA, USA) in Shanghai Applied Protein Technology Co., Ltd. (China). Both positive and negative ion modes were considered.

### 2.4. Proteomics profiling

Proteomics analysis was performed on the serum samples of patients with UC from Phase 2 in two groups: patients with both depression and anxiety, and patients without depression or anxiety. Each group consisted of three samples, with each sample pooled from six subjects. Serum pools were preprocessed and labeled using a tandem mass tag reagent (Thermo Fisher Scientific Inc., MA, USA). Liquid chromatography-tandem mass spectrometry analysis was performed on a Q-Exactive mass spectrometer (Thermo Fisher Scientific) coupled to Easy nLC, which was operated on a positive ion mode.

### 2.5. Animal experiments

Metabolites that decreased in patients with depression and anxiety (*p* < .05 for one phenotype and *p* < .1 for the other phenotype) were selected for the animal study. Male C57/B6J mice were used. Ten groups of six mice were investigated: the control group, dextran sulfate sodium (DSS)-treated group, and groups treated with four metabolites with/without DSS treatment. Another experiment enrolled 17 mice that were all treated with DSS to test the replicability of clinical data (referred to as replication experiment thereafter). During the metabolite treatment, the metabolite-treated mice were intragastrically administered with either 2ʹ-deoxy-D-ribose (20 mg/kg/day; Macklin, Shanghai, China), 4-hydroxybenzoate (10 mg/kg/day; Rhawn, Shanghai, China), L-pipecolic acid (10 mg/kg/day; Macklin), or hydroxyphenyllactic acid (25 mg/kg/day; Aladdin, Shanghai, China), while the mice in the control and DSS-treated groups were administered with saline. During model construction, the mice in the DSS treatment received drinking water containing 2.5% DSS for seven days to induce colitis.^29^ The metabolite treatment lasted for 21 days. During the last seven days, DSS was provided to construct colitis models for the metabolite-treated group with DSS. The metabolite-treated group without DSS only received the corresponding metabolites for 21 days. The mice in the replication experiment only received DSS for seven days. Behaviors were evaluated before and after the DSS treatment, with 1 h of rest between each. Altogether, three behavioral tests, namely, tail suspension test (TST), forced swimming test (FST), and open field test (OFT), were adopted to assess the depressive-like and anxious-like behaviors of mice (Supplementary Materials). The mice were sacrificed after the final behavioral test.

Disease activity index (DAI) scores were used to evaluate the severity of colitis in DSS-induced mice.

The serum of each mouse was separated from the whole blood. The hippocampus and prefrontal cortex were dissected from the brain tissue, homogenized on ice, and centrifuged at 3000 *g* for 20 min to obtain a supernatant of soluble proteins and metabolites. For metabolite-treated mice, IL-1β, IL-6, TNF-α, and dopamine levels in serum and hippocampus homogenates and serum IGHV3 and IGKV3 levels were measured using commercially available enzyme-linked immunosorbent assay (ELISA) kits (YIFEIXUE BIO TECH, Nanjing, China). IL-1β, IL-6, TNF-α, and LBP levels in serum and colon homogenates and LPS levels in the colon and hippocampus homogenates were measured by ELISA. The population of TMEM119 in the brain was measured by quantitative real-time PCR. The cecal samples were analyzed by 16S rRNA sequencing. The non-targeted metabolomics of the serum and brain homogenates (composed of hippocampus and prefrontal cortex) was profiled using the same method as that used for human samples.

### 2.6. Statistical analysis

Analyses and plotting were done with R version 4.0.1. Student’s t-test and general linear regression were chosen for the analyses of microbiota and metabolomics data. For proteomics, Student’s t-test was performed at a significance level of 0.05 and 95% confidence intervals. For microbiota data, a meta-analysis of patients with UC at Phase 1 and 2 was conducted based on the results of the Student’s t-test (Glass method) and general linear regression using the “meta” package implemented in R. Heterogeneity was assessed based on I^2^. A fixed model was considered if I^2^ < 50%, otherwise a random model was chosen. Microbial correlation was estimated using SparCC.^30^ Correlations were calculated in Spearman’s rank correlation, unless specified. A receiver operating curve (ROC) was used to compare the sensitivity and specificity of bacteria and metabolites in predicting phenotypes, with a prediction performance reflected by the area under the curve (AUC). RV coefficient analysis was performed by calculating the RV2 coefficient (modified RV coefficient) using the function “coeffRV” implemented in R package “FactoMineR.” A repeated measures analysis of variance was adopted to assess the results from ELISA. Multiple testing was either Bonferroni-corrected or corrected in false discovery rate (FDR).

## 3. Results

### 3.1. General characteristics

No significant difference was found in the general information of the studied groups, except for their body mass indices (Supplementary Table S1).

In patients with active UC, 55% (Phase 1) and 62% (Phase 2) also had depression, while 48% (Phase 1) and 53% (Phase 2) also had anxiety. None of the patients with UC took medication for their depression and anxiety symptoms in recent three months.

### 3.2. Fecal microbiota features in patients with active UC and depression/anxiety

**Figure 2.**
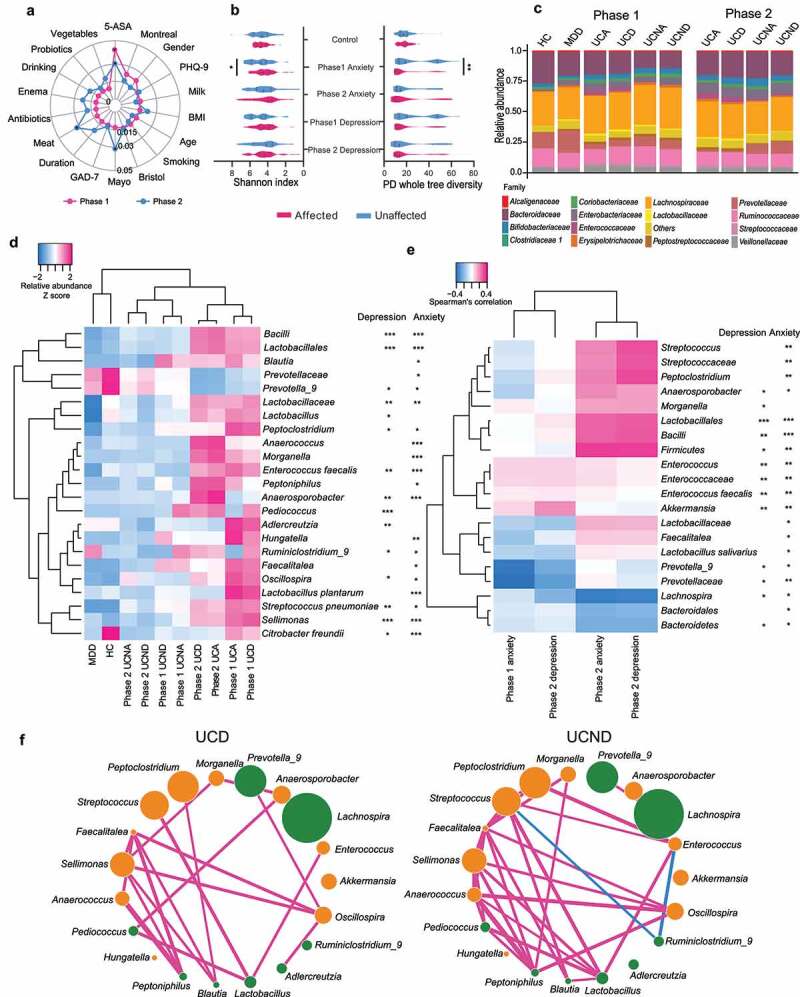
Changes in the gut microbiota of patients with UC (ulcerative colitis) and depression/anxiety. (a) Radar chart illustrates the top host factors associated with gut microbial structure in patients with UC. The gut microbial structure is illustrated by variations derived from between-sample unweighted UniFrac distances. Size effect and statistical significance were calculated by PERMANOVA (Adonis). The degree of impact on gut microbiota structure was defined into three degrees according to the value of R^2^: R^2^ < 0.015, low impact; 0.015≤ R^2^ < 0.03, moderate impact; R^2^ ≥ 0.03, high impact. Data for Phase 1 are illustrated in pink, while data for Phase 2 are in blue. (b) The α-diversity represented by Shannon index (left) and PD whole tree diversity (right) of gut microbiota in patients with UC are shown in violin plots, with significance determined by two-tailed Mann–Whitney U test. The groups with depression or anxiety are shown in pink violins, while those without are shown in blue violins. The control group includes healthy control (HC) and non-IBD patients with depression and anxiety (MDD). (c) The composition of gut microbiota at the family level in different groups at two phases is shown in stacked bar charts, with each family represented by a distinct color. The left six bars are for Phase 1 samples, while the right four bars are for Phase 2 samples. The UC group was divided into those with depression (UCD) and without depression (UCND) or those with anxiety (UCA) and without anxiety (UCNA). No significance was evaluated. (d) The relative abundances of the microbial taxa with statistical differences between UCD/UCA and UCND/UCNA groups based on Student’s t-test (*p* < .05) are shown in a heatmap. The abundance was standardized across all groups for each microbe. (e) Spearman’ correlation between depression/anxiety levels and the relative abundance of microbial taxa with statistical differences based on general linear regression (*p* < .05). The significance of association is indicated by asterisks as *p < .05, **p < .01, and ***p < .001. (f) Co-occurring (red line) and co-excluding (blue line) relationships of microbes in UCD and UCND. The edge width corresponds to the size of SparCC correlation coefficients. The edge color indicates the signs of SparCC correlation coefficients; red means positive correlation, while blue means negative correlation. Only connections with SparCC correlation coefficients |ρ|> 0.2 and *p* < .05 are shown. Enriched bacteria in UCD are illustrated in orange, while those enriched in UCND are in green

Among the host characteristics, depression levels (indicated by PHQ-9 scores) had a moderate impact (R^2^ > 0.015, Adonis test) on the gut microbiota structure of patients with UC (Figure 2(a)). Compared to patients with UC without depression or anxiety (UCND/UCNA), patients with UC and depression/anxiety (UCD/UCA) had lower fecal microbial community richness and diversity, with a low α-diversity at Phase 1 (represented by Shannon index and PD whole tree diversity; *p* = .07 for depression and *p* < .05 for anxiety; Figure 2(b), Supplementary Material). The composition of fecal microbiota differed among the groups (Figure 2(c), Supplementary Figure S1). For example, compared with UCND/UCNA, *Prevotellaceae* was increased, while *Bacteroidaceae, Lactobacillaceae, Enterococcaceae*, and *Streptococcaceae* were decreased in UCD/UCA.

To screen out the gut microbiota that were consistently associated with depression and anxiety phenotypes, both Student’s t-test and general linear regression were considered. The investigation at the taxonomy level revealed that four species, 15 genera, two families, one order, and one class showed differences in relative abundance between UCD/UCA and UCND/UCNA (Student’s t-test: *p* < .05; Figure 2(d)). The enrichment of *Sellimonas, Lactobacillales*, and *Bacilli* in both UCD and UCA was significant after multiple testing correction (*p* < 6.3 × 10^–5^ = 0.05/(197 × 2 × 2), considering 197 taxa, two phenotypes, and two statistical tests). In addition, compared with UCND/UCNA, *Sellimonas* was significantly enriched in UCD at both phases (*p* < .05). UCA harbored more *Anaerosporobacter* and *Anaerococcus*, while UCD harbored more *Pediococcus* (Bonferroni-corrected; *p* < .05). The regression with phenotypic scores suggested that 20 taxa were associated with depression/anxiety levels (Figure 2(e)). *Bacilli* and *Lactobacillales* were positively correlated with anxiety levels after multiple testing correction. Ten taxa were suggested to be associated with depression/anxiety based on both statistical tests, and five of them were shared by both depression and anxiety, namely, *Enterococcus faecalis, Anaerosporobacter, Prevotella_9, Bacilli*, and *Lactobacillales*. Based on either test, most of these taxa had an increased abundance in UCD/UCA; they mainly belong to *Lactobacillales* and *Clostridiales* (both belong to *Firmicutes*), and these associated genera were concentrated in two families, *Lactobacillaceae* and *Lachnospiraceae*. The increase in *Sellimonas* and decrease in *Prevotella_9* were also observed in the MDD group compared to the control group (Supplementary Table S2). *Lachnospira* and *Anaerosporobacter* were associated with neither UC nor pure depression/anxiety phenotypes, indicating their unique roles in UC accompanied by depression/anxiety (Supplementary Table S2&S3). ROC analysis suggested that some bacteria, such as *Prevotella_9, Dorea*, and *Collinsella*, had fair diagnostic potentials for classifying UC-related depression/anxiety phenotypes (AUC > 70%; Supplementary Figure S2).

To explore the functional implications of microbial shift that drives patients with UC to be depressive and anxious, microbial functional pathways were investigated using PICRUSt version 1.1.4. Cysteine and methionine metabolism were down-regulated in UCA/UCD at Phase 2 as compared with UCNA/UCND, but this was unrelated to UC itself, as suggested by the insignificant difference between patients with UC and the control (Supplementary Table S4). It was also negatively correlated with the abundance of *Bacilli* and *Prevotella_9* (Spearman’s rank correlation test, *p* < .05; Supplementary Figure S3). Tetracycline biosynthesis and Vitamin B6 metabolism were significantly down-regulated in UCA/UCD as compared with UCNA/UCND at Phase 2 and were associated with UC phenotypes (Supplementary Table S4); these two pathways were significantly correlated with the abundance of *Blautia, Bacilli, Prevotella*_*9*, and *Hungatella*. In addition, Vitamin B6 metabolism was associated with the abundance of *Sellimonas* (Supplementary Figure S3).

As a functional community, gut microorganisms dynamically interact with each other to form a topological network. The gut microbial network of UCD harbored fewer connections than that of UCND (figure 2(f), Supplementary Figure S4). Especially, the co-occurring network of *Streptococcus, Enterococcus, Peptoclostridium*, and *Faecalitalea* disappeared in the feces of UCD/UCA, but *Prevotella_9* developed new co-occurring connections with other bacteria (figure 2(f)).

### 3.3. Metabolomic signatures of patients with active UC and depression/anxiety

**Figure 3.**
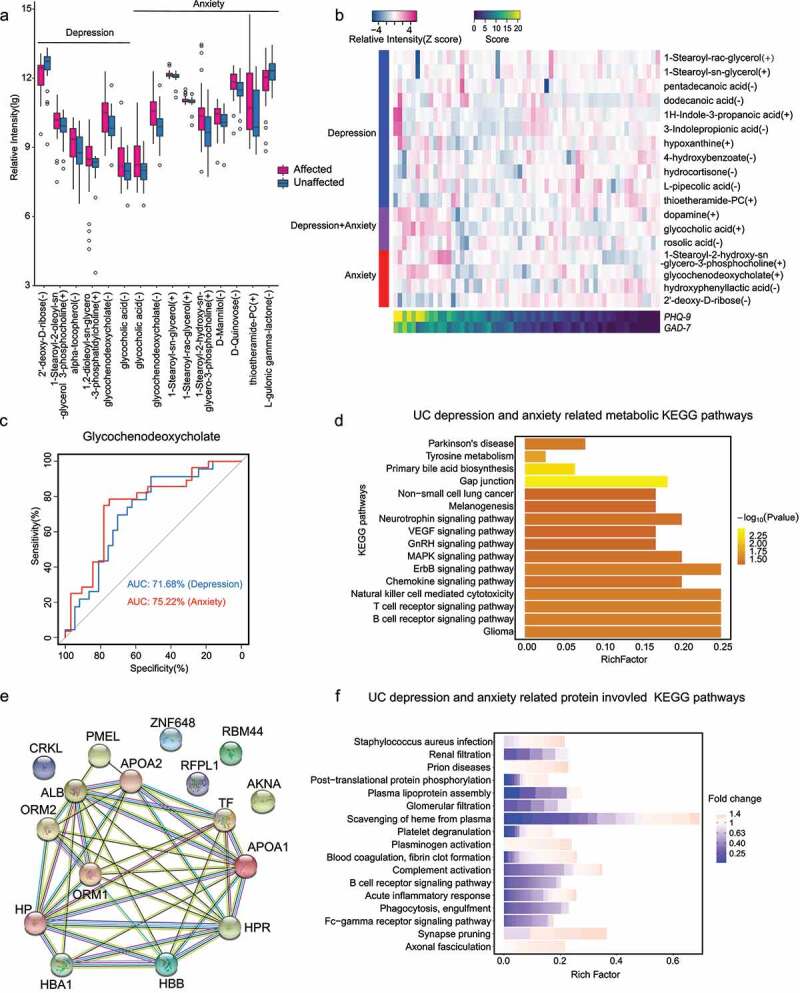
Serum metabolomics and proteomics alterations in patients with UC and depression/anxiety in Phase 2 (n = 60). (a) Differential metabolites between UCD/UCA and UCND/UCNA (by Student’s t-test) and VIP > 1 are illustrated in a box plot. (b) The relative abundance of the metabolites (row) significantly associated with UCD/UCA based on general linear regression is shown in a heatmap for each subject (column) and scaled across subject. (c) Diagnostic ability of the top metabolite associated with depression and anxiety in patients with UC based on ROC (receiver operating characteristic). (d) The metabolomic KEGG pathways with significant differences in both UCD and UCA are shown. (e) The protein-protein interaction network shows the significantly regulated proteins associated with depression and anxiety in patients with UC (Student’s t-test, *p* < .05, fold change>3). (f) Selective proteomic pathways with statistical differences are shown. The bar color represents the fold change of each significantly regulated genes in the pathway. The fold change is the expression ratio of UCD/UCA group to UCND/UCNA group

Compared with UCNA/UCND, UCA/UCD harbored more glycocholic acid and glycochenodeoxycholate (VIP > 1, fold change > 1.5; Student’s t-test and general linear regression; *p* < .05; Figures 3(a,b)). The ROC analysis showed that glycochenodeoxycholate alone had a fair prediction performance on UC-related depression and anxiety (AUC = 75%, Figure 3(c)), while 1-stearoyl-2-hydroxy-sn-glycero-3-phosphocholine was positively associated with anxiety based on both analyses and had a potential to be a predictor of anxiety levels (Figures 3(a,b); Supplementary Figure S5). Besides, 2ʹ-deoxy-D-ribose, 1-stearoyl-sn-glycerol,1-stearoyl-rac-glycerol, and thioetheramide-PC were all revealed by both analyses to be related with depression and anxiety in patients with UC (Figures 3(a,b)). It should be noted that dopamine, a well-known neurotransmitter related to the reward system in the brain, was positively associated with both anxiety and depression levels in patients with UC (Figure 3(b), Supplementary Figure S5).

These phenotype-associated metabolites were enriched in 16 KEGG pathways, mainly involving immunological and neurological functions (Figure 3(d), Supplementary Table S5). Primary bile acid biosynthesis (involving glycocholic acid and glycochenodeoxycholate) and gap junctions (involving dopamine) were associated with both UCD and UCA. It is noteworthy that tyrosine metabolism, a well-known pathway involved in psychological disorders, was disturbed in UCA (Figure 3(d)). In this pathway, hydroxyphenyllactic acid and dopamine were also associated with anxiety levels in patients with UC (Figure 3(b)).

### 3.4. Proteomic features in patients with active UC and depression/anxiety

The serum proteome profiling discovered 132 proteins that were significantly different between UCA/UCD and UCNA/UCND (*p* < .05; Supplementary Table S6); 26 of them, mainly immunoglobulin proteins, passed multiple testing corrections. Eighteen proteins were significantly down-regulated by over three times, and they closely interact with each other (Figure 3(e), Supplementary Table S7). These associated proteins were significantly (*q* < 0.05) enriched in pathways involving down-regulated inflammatory responses, phosphatidylcholine and cholesterol metabolic processes, and phagocytosis but upregulated blood coagulation (figure 3(f)). The upregulated blood coagulation was consistent with the clinical manifestations of UC.

### 3.5. Integrative crosstalk of multi-omics

**Figure 4.**
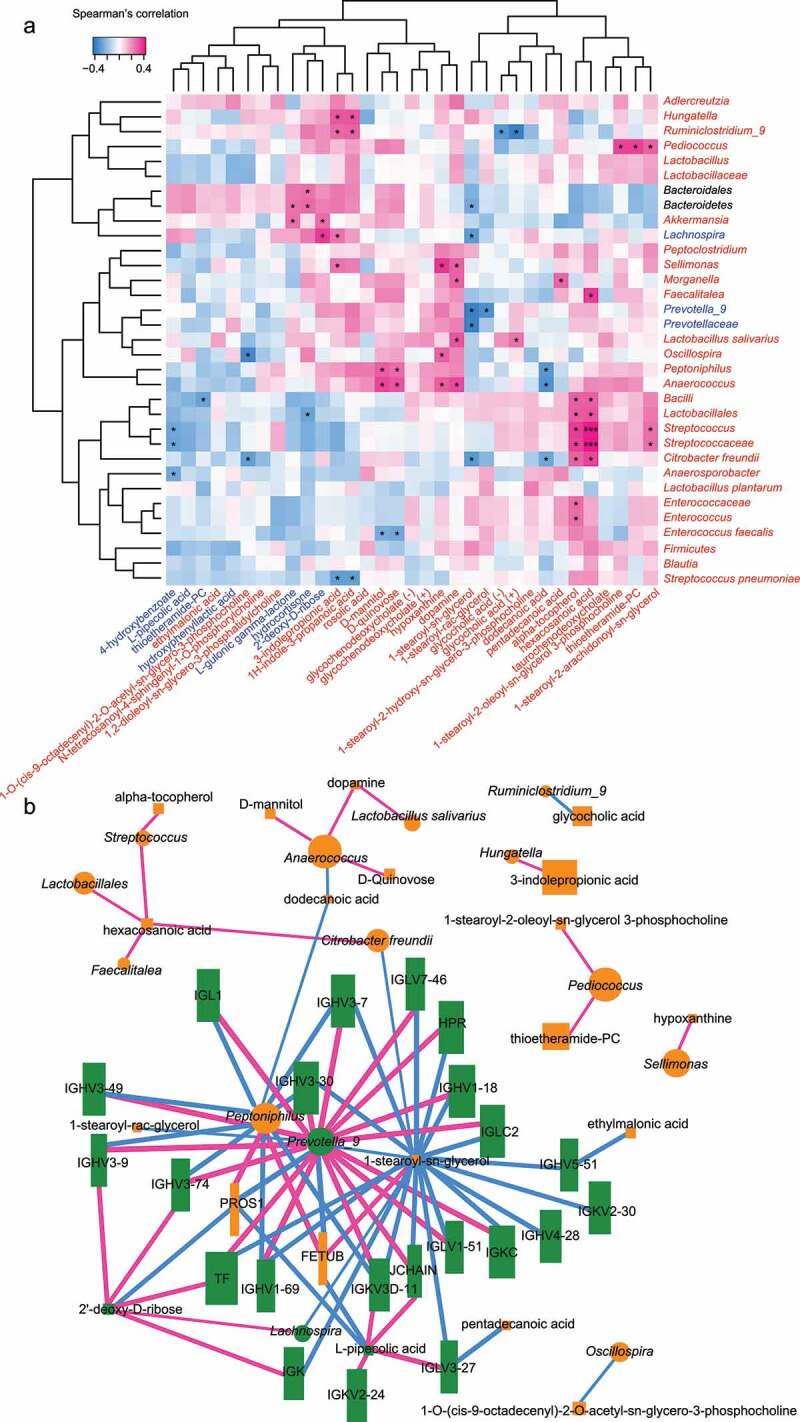
Integrative data crosstalk of multi-omics. (a) Spearman’s rank correlation between phenotype-associated microbiota and metabolites. The red font represents a higher abundance in UCD/UCA, while the blue font represents a lower abundance. The black font indicates an opposite trend in the two phases. Significance was indicated as *p < .05, **p < .01, and ***p < .001. (b) The network diagram shows the interaction among microbiota, metabolites, and proteins. The color of the node represents the correlation between bacteria (or metabolite, protein) and phenotype; abundant bacteria in UC depression and anxiety are shown in orange, while abundant bacteria in UC non-depression and anxiety are shown in green. The color of the edges represents the correlation between two nodes; a positive correlation is shown in pink, while a negative correlation is shown in blue

The crosstalk among gut microbiota, serum metabolome, and clinical phenotypes was quantified by calculating the RV coefficients and Spearman’s correlation coefficients. The gut microbiota shared 18% variation with serum metabolome (*p* = .13) and 17% with participants’ lifestyle (*p* = .018), suggesting a mild similarity between the multi-omics data (Supplementary Figure S6). There was a strong correlation between specific microbes, serum metabolites, and proteins associated with UCD/UCA (Figure 4(a), Supplementary Figure S7, S8). *Streptococcus* and hexacosanoic acid have a significant positive correlation (*p* < .001, Spearman’s rank correlation test). *Prevotella_9* was negatively correlated with metabolites 1-stearoyl-sn-glycerol and 1-stearoyl-rac-glycerol. UCD/UCA displayed a decreased amount of beneficial *Lachnospira* and 2ʹ-deoxy-D-ribose but had an increased level of *Sellimonas*, hypoxanthine, dopamine, and 3-indolepropionic acid (*p* < .05, Spearman’s rank correlation test). Furthermore, the combination of these bacteria and metabolites could fairly predict the depression or anxiety levels of patients with UC (AUC> 70%, Supplementary Figure S9).

The significantly correlated gut microbiota, serum metabolites, and proteins formed a comprehensive network (Figure 4(b)), centering on *Peptoniphilus, Prevotella_9*, and 1-stearoyl-sn-glycerol. This network center was surrounded by *PROS1, FETUB, JCHAIN*, and a set of immunoglobulin proteins, which were reduced in UCD. Some of these immune-related proteins were further associated with potentially beneficial 2ʹ-deoxy-D-ribose and L-pipecolic acid. There were two additional small network clusters – one was centered on *Anaerococcus* and dopamine, while the other was centered on hexacosanoic acid and *Streptococcus.*

The gut microbiota and the host’s serum share pathways of *Staphylococcus aureus* infection. Serum metabolites and proteins were involved in similar pathways related to cytokine signaling and immune responses, since they overlapped on the B-cell receptor signaling pathway (Figures 3(d,f)). All these indicated a disturbance in immune response against bacteria in UCD/UCA.

### 3.6. Effects of candidate metabolites on mice induced with colitis

**Figure 5.**
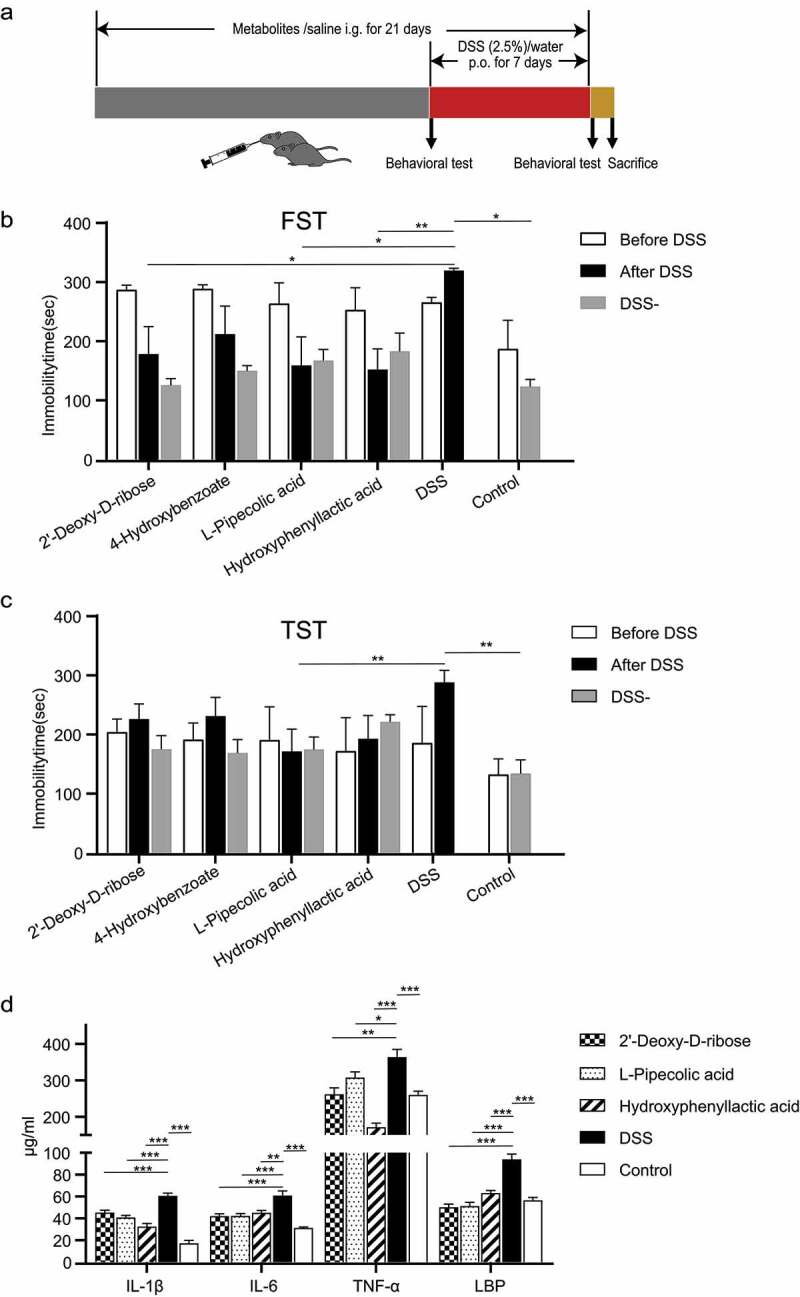
(a)Work flow illustrating the experiment studying treatment with selected metabolites in mice to rescue their depressive and anxious like behaviors. i.g., intragastric; p.o., oral. Three behavioral tests were given, namely, forced swimming test (FST) and tail suspension test (TST) for measurement of depressive-like behavior, and open field test (OFT) for measurement of anxious-like behavior. (b-c) Effect of metabolites treatment on the immobility time in FST (b) and TST (c) in DSS-treated and healthy mice. (d) Quantification of three pro-inflammatory cytokines (IL-6, IL-1β, TNF-α) and LBP in the colon of DSS-treated and healthy mice under intervention of different metabolites. Bars represent means ± standard error of mean. Significance levels are indicated as * *p* < .05, ** *p* < .01, and *** *p* < .001

The metabolites that were suggestively significantly reduced in both UCD and UCA, namely, 2ʹ-deoxy-D-ribose, 4-hydroxybenzoate, L-pipecolic acid, and hydroxyphenyllactic acid, were selected to investigate their impact on depressive-like and anxious-like behaviors of mice with colitis (Figure 5(a)). Compared with the control group, DSS-treated mice had a longer immobility time in FST (*p* = .034, Student’s t-test, Figure 5(b)) and TST (*p* = .0015, Student’s t-test, Figure 5(c)), and had weight loss, hematochezia, loose stool, and shortened colon length. By comparing the central residence time and movement distance in OFT, no significant anxious-like behavior was observed in DSS-treated mice (*p* > .05, Student’s t-test; Supplementary Figure S10). These experimental results suggest the successful induction of depressive-like behavior and colitis in mice by 2.5% DSS treatment for seven days. Compared to those with little depressive-like behaviors, the mice that displayed more depressive-like behaviors after the DSS treatment showed no difference based on DAI scores or histopathological manifestations in the rectum. This suggests no significant difference in the severity of colitis, which was consisted with clinical findings. Compared to DSS-treated mice without deteriorated depressive behaviors, DSS-treated mice with more depressive behaviors showed increased serum IL-1β, IL-6, and TNF-α levels, elevated intestinal mucosal IL-6, TNF-α, and LPS levels, upregulated LPS and LBP levels in the brain (*p* > .05, Student’s t-test; Supplementary Figure S11A). 123 serum metabolites identified in the mice model were also present in human serum metabolomic data, and 71 had the same trend as in patients with UC in the comparison of depression vs. non-depression. Besides, 68 metabolites in the brain tissue that coincided with human serum metabolomic data were identified, and 42 of which displayed the same trend as that in patients with UC in the comparison of depression vs. non-depression. Compared with DSS-treated mice without depressive-like behaviors, DSS-treated mice with more depressive-like behaviors had lower brain and serum levels of L-pipecolic acid and lower serum hydroxyphenyllactic acid levels, which were consistent with the findings in human metabolomics.

The prophylactic administration of L-pipecolic acid in DSS-treated mice significantly reduced the immobility time in FST and TST (Figures 5(b,c)). Additionally, 2ʹ-deoxy-D-ribose and hydroxyphenyllactic acid could reduce the immobility time in FST (Figure 5(b)). However, only the administration of these metabolites had no significant influence on the behavioral performance (Figure 5(b)), body weight, and colon length of healthy mice.

We further depict the gut microbiota in these mice with colitis accompanied with depressive-like behaviors. As observed in human subjects, a few microbes, such as *Enterococcus* and *Streptococcus*, were enriched in mice with colitis, while microbes, such as *Dorea*, were decreased. Some of these affected microbes, such as *Dorea*, could be rescued by applying the four beneficial metabolites, but other microbes were irresponsive to metabolite treatment (Supplementary Figure S12).

As observed in UCD/UCA, dopamine was significantly elevated in the blood and brain of DSS-treated mice, and it was significantly alleviated by the administration of 2ʹ-deoxy-D-ribose and L-pipecolic acid (*p* < .005, Supplementary Figure S13A, Supplementary Results).

Since both UC and depression/anxiety are associated with chronic inflammation, changes in certain immune-related proteins and several pro-inflammatory cytokines in the serum, colon, and hippocampus (a publicly reported depression-related brain region) were further examined during colitis induction and metabolite treatment. Two immune-related proteins, IGHV3 and IGKV3, were selected for the investigation because most of their family members (e.g., IGHV3-9 and IGKV3D-11) were significantly decreased in UCD/UCA. In accordance with the findings in humans, IGHV3 and IGKV3 were lowered when colitis was induced in mice, and they were significantly elevated when mice were treated with either 2ʹ-deoxy-D-ribose, L-pipecolic acid, or hydroxyphenyllactic acid (Supplementary Figure S14, Supplementary Results). Furthermore, IGHV3 was only specifically enriched in the 2ʹ-deoxy-D-ribose-treated group, while its level was specifically significantly associated with L-pipecolic acid administration, consistent with the findings in humans. Mouse model with DSS-induced colitis displayed an extensive inflammatory state, manifested by a significant elevation of pro-inflammatory cytokines such as IL-6 in the blood, colon, and brain (Figure 5(d), Supplementary Figure S13), and elevated LBP in the colon (Figure 5(d)). This inflammatory state could be mitigated by prophylactic administration of 2ʹ-deoxy-D-ribose and L-pipecolic acid, as these two can reduce the levels of pro-inflammatory cytokines and LBP in the colon, and downregulated serum levels of IL-6, TNF-α, and dopamine in healthy mice (Figure 5(d), Supplementary Figure S13, Supplementary Results). All these evidences support the finding that the prophylactic use of these beneficial metabolites reduced the depressive-like behavior and ameliorated the inflammatory response in mice with colitis.

## 4. Discussion

Nowadays, the use of multi-omics approach is getting popular and common in studying a complex disease and the relationship between two concomitant diseases.^23,31^ However, few attempts have been tried on UC-related disorders. Previously, only one Swiss cohort study on 171 patients with IBD in remission described the relationship between impaired psychological function and gut microbiota.^27^ However, there is no study on gut microbiota, metabolomics, and other characteristics of patients with active UC (a type of IBD). Herein, we report the molecular signatures of active UC with depression and anxiety in terms of gut microbiota, serum metabolomics, and serum proteomics using a meta-analysis approach and two additional control groups (240 participants in total). It was found that the prophylactic administration of 2ʹ-deoxy-D-ribose and L-pipecolic acid, which were enriched in UCND/UCNA as compared to UCD/UCA, could significantly mitigate the depressive-like behaviors of mice with colitis.

Compared to UCND/UCNA, UCD/UCA harbored less *Prevotella_9* in the feces. Consistently, *Prevotella* is associated with lower levels of depression and anxiety in patients with IBD and less depression and anxiety mood in the healthy population.^27,32–34^ *Prevotella* was reported to participate in emotion-related tryptophan and glutamate synthesis.^33^ Serum L-tryptophan and L-glutamate levels were lower in UCD/UCA. In the current study, bile acid derivatives, glycocholic acid, and glycochenodeoxycholate were elevated, and the bile acid synthesis pathway was significantly changed in UCD/UCA. As bile acids can be transformed by gut bacteria to affect host metabolism and innate immunity, bile acids possibly have a role in the mechanism of UC accompanied with depression/anxiety.^35^ A co-occurring bacterial relationship, whose bacteria were reported to modulate immune response,^36^ was weakened in UCD compared to that in UCND. This indicates that more attention should be paid to co-occurring or co-excluding connections among bacteria in future studies.

Patients with active UC and depression/anxiety shared some similar gut microbes with non-IBD patients with depression and anxiety, such as *Prevotella_9* and *Sellimonas*. These might contribute to the comorbidity of UC and depression/anxiety. However, UCD/UCA had a set of specific gut microbiota, such as *Lachnospira*, which was not enriched in UCND. It is noteworthy that UCD/UCA (as compared to UCND/UCNA) shared much more characteristic microbiota with UC (as compared to the healthy control) than with MDD (as compared to healthy control), indicating a closer mechanism to UC rather than MDD in terms of gut microbiota.

Meanwhile, 2ʹ-deoxy-D-ribose is a DNA backbone, and it could be consumed and synthesized by various gut bacteria.^37–39^ It plays a role in resisting apoptosis and in regulating the host’s immune response.^40–42^ However, its function in the nervous system is still unclear. Since it was significantly decreased in UCD/UCA, and its supplementation in mice could significantly mitigate their DSS-induced depressive-like behaviors, it is worthwhile to investigate the molecular mechanism of 2ʹ-deoxy-D-ribose in UCD/UCA. Another metabolite that decreased in UCD/CUA is L-pipecolic acid, which is an intermediate metabolite of L-lysine in the brain, and it could activate GABA receptors.^43,44^ Both GABA and L-pipecolic acid were reduced in the blood of mice with colitis and depressive-like behaviors, which could be alleviated by L-pipecolic acid supplementation. Major depression and anxiety disorders share a GABAergic deficit as a common pathophysiology, L-pipecolic acid can potentially relieve depression and anxiety via the GABAergic system.^45^ The intervention of 2ʹ-deoxy-D-ribose and L-pipecolic acid in DSS-treated mice restored the serum IGHV3 and IGKV3 levels, respectively, and decreased the pro-inflammatory cytokines systematically (in the blood) and locally (in the hippocampus). As the serum levels of these two metabolites were significantly correlated with the serum levels of the two immunoglobulins in patients with UC, the two beneficial metabolites possibly have a potential role in the host immune response of patients with UC and depression/anxiety.

**Figure 6.**
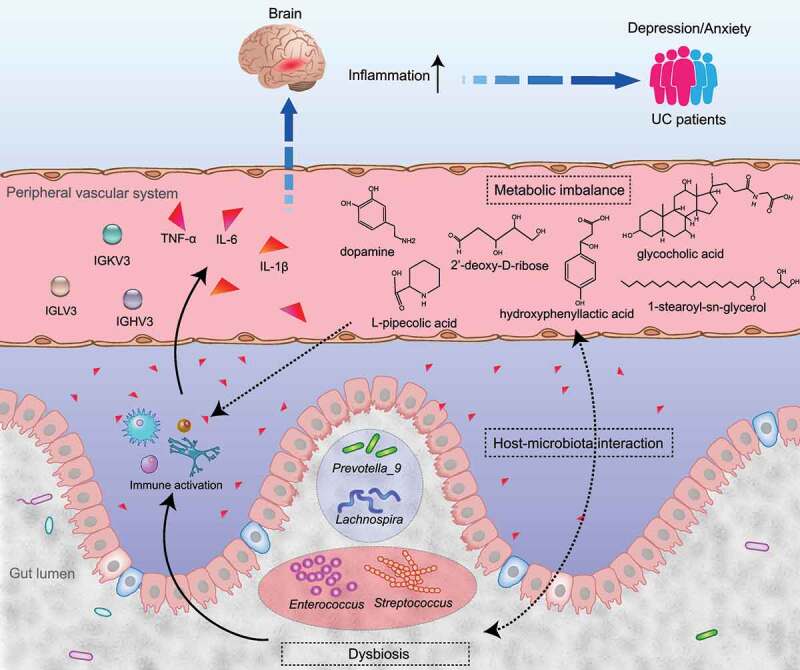
Suggested model illustrating the network of gut microbiota, serum metabolites, and proteins in the pathogenesis of UC-related depression and anxiety. Patients with active UC have a disturbed gut microbiota. This disorganized gut microbiota interacts with blood metabolites through host-microbiota interaction. Together, they affect the soluble proteins in the blood, increasing the systematic inflammation and decreasing the host’s immune response (manifested by reduced immune-related proteins). The increased inflammatory state is transmitted to the brain (especially in the hippocampus, which is involved in depression and anxiety), influencing the mood state. This leads to depression and anxiety in patients with UC. Solid lines show the links suggested by previous studies. Dashed lines indicate the possible connections that can be inferred from our findings

The results of both metabolomic and proteomic pathway analysis and the amount of immunoglobulin changes suggest the involvement of immune response in the development of depression and anxiety in patients with active UC (Figures 3, 6). This increase in inflammation was supported by the increased LPS level in the colon and brain, elevated pro-inflammatory cytokine levels in the colon, blood, and brain, and decreased serum immunoglobulin levels in the mice with colitis and depressive-like behaviors. Further studies are needed to validate and characterize the role of inflammation in the development of UC-related depression and anxiety.

Numerous bacteria, such as *Peptoniphilus* and *Ruminococcus*, that were discovered to be associated with UC-related depression and anxiety could be influenced by medication with L-dopa (a precursor of dopamine), suggesting a potential association between these bacteria and dopamine metabolism.^46^ In this study, dopamine was found to be significantly elevated in UCD/UCA compared to UCND/UCNA. This trend was replicated in mouse models, similar to previous studies where systemic inflammation that originated from the gut induces a dopamine increase.^47–50^ On the other hand, the administration of beneficial metabolites, such as 2ʹ-deoxy-D-ribose and L-pipecolic acid, could significantly reduce the elevated dopamine levels in the serum and hippocampus of mice with colitis.

In this study, the prevalence of depression and anxiety in patients with active UC reached over 50%, which was consistent with the results of a recent meta-study.^5^ However, anti-depressant/anxiolytic drug use in patients with UC is rare, and most patients will not actively report their emotional states for medical assistance. Thus, clinicians should be aware of the possible depression and anxiety in patients with UC to provide treatments accordingly in time.

There are several limitations in this study. First, the small sample size may produce large variations. Second, it is a cross-sectional observational study in terms of gut microbiota, which could not reveal a causal relationship, and there was a lack of dissection in microbe-host interactions. Future validation using germfree animal models is needed. Third, the assessment of depression and anxiety symptoms through questionnaires is not accurate for clinical diagnosis. Fourth, this study only included patients with active UC, which was not enough to represent all IBD or other diseases with a feature of chronic stress.

In conclusion, this study reveals the gut microbiota and metabolome characteristics in patients with active UC and depression/anxiety, and an interaction between them was suggested in correlation analyses. Patients with active UC accompanied by depression and anxiety had lower fecal microbial community richness and diversity, with disorganized gut microbiota and disturbed metabolism; these could reduce immune-related proteins in the blood and increase systematic inflammation. The increased inflammatory state could be transmitted to the brain and influence the mood state, which might explain the development of depression and anxiety in patients with UC (Figure 6). It suggests that restoring and regulating the disturbed gut microbiota and metabolome by targeting these characteristic microbes and metabolites could help in the clinical intervention of depression and anxiety for patients with active UC.

## Supplementary Material

Supplemental Material

## Data Availability

The microbial and metabolomic raw data reported in this paper have been deposited in the China National Center for Bioinformation (https://ngdc.cncb.ac.cn) and the accession numbers are HRA001244 (human microbiota), CRA004834 (animal microbiota), OMIX553 (human metabonomics), OMIX712 (animal metabonomics).

## Preprint information

This manuscript has been posted to a preprint server Research Square (https://www.researchsquare.com/) with DOI: https://doi.org/10.21203/rs.3.rs-49500/v1
