# Supplementary material for: Depression and anxiety in patients with active ulcerative colitis: crosstalk of gut microbiota, metabolomics and proteomics
Source: Gut Microbes. 2021 Nov 21;13(1):1987779. doi: 10.1080/19490976.2021.1987779 (PMC8632339; doi:10.1080/19490976.2021.1987779)
Supplement: Supplemental Material [file KGMI_A_1987779_SM8304.zip › Supplementary information/Supplementary material (1).docx]

**Supplementary Materials and methods**

*Study design and population*

This study was performed in accordance with the principle of the Helsinki Declaration II. Subjects in Phase 1 and the two control cohorts were recruited in 2018, while subjects in Phase 2 were recruited in 2019. Demographic information, mental health measurements, dietary habits, and Bristol stool scores of all subjects were collected. In addition, patients with ulcerative colitis (UC) also reported their disease type, severity of disease, duration of disease, medication history and laboratory examination results. Patients with active UC were recruited from the outpatient department and gastroenterology department of the Jiangsu Province Hospital of Chinese Medicine. All participants were diagnosed with UC, with active UC defined as Mayo score≥ three, and Mayo endoscopy score≥ one. The Mayo score is a composite of four subscores, namely, stool frequency subscore, rectal bleeding subscore, endoscopy subscore, and physician's global assessment subscore. It ranges from 0 to 12, with each of the four subscores ranging from 0 to 3. The higher the score, the more severe the UC. Scores 0-2, 3-5, 6-10, and 11-12, represent disease remission, mild, moderate, and severe, respectively. Patients with depression and anxiety but without inflammatory bowel disease (IBD) were recruited from the psychological outpatient department of the Jiangsu Province Hospital of Chinese Medicine. These patients presented at the hospital because of subjective complain of anxiety, depression, or other emotional disturbance symptoms. These patients were asked to answer PHQ-9 and GAD-7 questionnaires, as well as an IBD-related general questionnaire. IBD-related general questionnaire was designed to screen for gastrointestinal symptoms and IBD history of the patients or their family members, with two questions as following: (1) Have you and /or your close relatives being diagnosed of any gastrointestinal disease? (2) Do you have any gastrointestinal symptom such as rectal bleeding, constipation, or diarrhoea? If the patients met Patient Health Questionnaire-9 (PHQ-9) scores over four, Generalized Anxiety Disorder Scale (GAD-7) scores over four, and no IBD-related symptom, they would be asked if they would like to participate in this study, and an informed consent would be given to them with instructions of subsequent sample collection. Healthy subjects matched in age and sex and not accompanied by any mental disorders including major depression and anxiety disorders were recruited at the **Medical examination center** of the Jiangsu Province Hospital of Chinese Medicine. They also completed PHQ-9 and GAD-7 questionnaires, and only those with both PHQ-9 scores and GAD-7 scores less than five were included in the subsequent study. All the participants (including patients with UC, patients with depression and anxiety but without IBD, and healthy subjects) were screened for any identified mental health problems other than depression and anxiety disorders, such as schizophrenia and autism, by answering the following questions implemented in the questionnaire: (1) Have you ever been diagnosed with any kind of mental disorder other than depression or anxiety, such as schizophrenia, bipolar disorder, autism, obsessive-compulsive disorder, attention deficit hyperactivity disorder, etc.? (2) Do your immediate family members have any history of mental illness? Those patients reporting themselves or relatives being diagnosed with mental health problems other than depression and anxiety disorders were excluded.

Moreover, malignant tumors and autoimmune diseases in patients with depression and anxiety but without IBD and in healthy subjects were excluded through interviews and questionnaires. Patients who were unable to understand or provide informed consent, and those who did not have a confirmed diagnosis of IBD in their medical records were excluded. Consented patients provided demographic information. All groups of subjects did not use any form of antibiotics within four weeks before sample collection. Patients with UC did not receive enema treatment within half a month, but other therapeutic drugs were not restricted. Patients either with systematic infectious diseases, major gastrointestinal surgery history, or mental disorders except depression and anxiety were excluded. Diet was not controlled.

*Definition of anxiety and depression levels*

PHQ-9 is a nine-item, self-reported questionnaire that assesses symptoms of depression^1^. Each item of the PHQ-9 maps onto one DSM-IV major depression criterion, PHQ-9 has high specificity and sensitivity in the screening of depression. The total score of PHQ-9 ranges between 0 and 27, and the critical scores are 5, 10, 15, and 20. Scores of 0-4, 5-9, 10-14, 15-19, and 20-27 represent no, mild, moderate, moderate to severe, and severe depression, respectively.

The seven-item GAD-7 is developed as a screening tool for detecting anxiety levels in patients and has become a widely used measure for adults.^2^ It includes seven questions, with a total score of 0-21, and critical scores at 5, 10, 14, and 19. Scores of 0-4, 5-9, 10-13, 14-18, and 19-21 represent no, mild, moderate, moderate to severe, and severe anxiety, respectively.

*Sample preparation*

Every participant received a stool sample collection kit (Beijing Allwegene Technology Co., Ltd., China) with an instruction of the collection procedure explaining the operation details so as to minimize possible contamination. Fecal samples were collected and transferred to -80℃ for storage within two hours after collection until subsequent sequencing procedure. Five ml of fasting serum from all patients with UC in the replication cohort were collected and stored at -80℃ for subsequent non-target serum metabolomics and serum proteomics analyses.

*High-throughput 16S rRNA gene sequencing*

DNA collected by kits (Beijing Allwegene Technology) was extracted using an E.Z.N.A.R Stool DNA Kit (Omega Bio-tek, Norcross, GA, U.S.A.). Purity and quality of the genomic DNA were checked on the 0.8% agarose gels.

The V3-V4 hypervariable region of bacterial 16S rRNA gene was amplified by polymerase chain reaction (PCR), with the primers as 338F (ACTCCTACGGGAGGCAGCAG) and 806R (GGACTACHVGGGTWTCTAAT)^3^. For each fecal sample, an eight-digit barcode sequence was added to the 5' end of the forward and reverse primers (Beijing Allwegene Technology). PCR was carried out on a Mastercycler Gradient (Eppendorf, Germany) using 25 μl reaction volumes, containing 12.5 μl KAPA 2G Robust Hot Start Ready Mix, 1 µl forward primer (5 µM), 1 µl reverse primer (5 µM), 5 µl DNA (total template quantity was 30 ng), and 5.5 µl H_2_O. Cycling parameters were 95℃ for 5 min, followed by 28 cycles of 95℃ for 45 s, 55℃ for 50 s, and 72℃ for 45 s, with a final extension at 72℃ for 10 min. PCR products were purified using a QIAquick Gel Extraction Kit (QIAGEN, Germany), quantified using real-time quantitative PCR, and sequenced at Beijing Allwegene Technology. Deep sequencing was performed on a Miseq platform (Illumina, USA) and then analysed using Illumina Analysis Pipeline Version 2.6.

Raw data were first screened and reads were removed from subsequent analyses if they either were shorter than 200 bp, had a low quality score (≤ 20), contained ambiguous bases, or did not exactly match to primer sequences and barcode tags. Qualified reads were then distinguished using the sample-specific barcode sequences, trimmed with Illumina Analysis Pipeline Version 2.6, and then analysed using QIIME. Sequences were clustered into OTUs at a similarity level of 97%^4^, to generate rarefaction curves and to calculate the richness and diversity indices. The Ribosomal Database Project Classifier tool was used to classify all sequences into taxonomic groups, according to the SILVA 128 database.^5^

To examine similarity between different samples, clustering analysis and principal component analysis (PCA) were performed based on the OTU information from each sample using R.^6^ The evolution distances between microbial communities for each sample were calculated using the unweighted unifrac algorithm and represented by an Unweighted Pair Group Method with Arithmetic Mean clustering tree describing the dissimilarity (1– similarity) between multiple samples.^7^ A Newick-formatted tree file was generated through this analysis.

*Metabolomics profiling of human serum samples*

Fasting blood samples were collected in 5 ml vacutainer tubes, then centrifuged for 15 min at 1500 *g*, 4℃ to collect the plasma. Each aliquot (150 μl) of the plasma sample was stored at -80℃ until analysis. The plasma samples were thawed at 4℃ and 100 μl aliquots were mixed with 400 μl of cold methanol/acetonitrile (1:1, v/v) to remove the proteins. The mixture was centrifuged for 15 min at 14000 *g*, 4℃. The supernatant was dried in a vacuum centrifuge. For Liquid chromatography tandem mass spectrometry (LC-MS/MS) analysis, samples were re-dissolved in 100 μl acetonitrile/water (1:1, v/v). To monitor the stability and repeatability of instrument analysis, quality control (QC) samples were prepared by pooling 10 μl of each sample and analysed together with the other samples. The QC samples were inserted regularly and analysed in every five samples.

Metabolomic profiling of samples was performed on an Agilent 1290 Infinity LC system (Agilent Technologies, California, USA) coupled with an AB SCIEX Triple TOF 6600 System (AB SCIEX, MA, USA) in Shanghai Applied Protein Technology Co., Ltd (Shanghai, China). Chromatographic separation was implemented on ACQUITY UPLC BEH Amide 1.7 µm (2.1 × 100 mm) columns for both positive and negative modes. The column temperature was set at 25℃. The mobile phase contained an aqueous solution of 25 mM ammonium acetate and 25 mM ammonium hydroxide (A) and acetonitrile (B) mixture. The gradient was 95% B and 5% A for 1 min, with a linear reduction to 65% B and 35% A over 13 min, a reduction to 40% B and 60% A over 2 min, maintenance for 2 min and an increase to 95% B and 5% A over 0.1 min, with a 5-min re-equilibration period. The delivery flow rate was 300 μl/min, and 2 μl aliquot of each sample was injected onto the column.

For mass spectrometric (MS) detection, the following electrospray-ionization source conditions were used: ion source gas 1 (Gas1) of 60 psi, ion source gas 2 (Gas2) of 60 psi, curtain gas (CUR) of 30 psi, source temperature of 600℃, and ion spray voltage floating of ± 5500 V. In the MS-only acquisition, the instrument was set to acquire data over the m/z range of 60-1000 Da, and the accumulation time for the time-of-flight tandem mass spectrometry (TOF-MS) scan was set to 0.20 s/spectrum. For auto MS/MS acquisition, the instrument was set to acquire data over the m/z range of 25-1000 Da, and the accumulation time for the product ion scan was set to 0.05 s/ spectrum. The product ion scan was recorded using information-dependent acquisition with a high-sensitivity mode. The parameters were as follows: collision energy (CE): fixed at 35 V± 15 eV; declustering potential (DP): 60 V (+) and −60 V (−); exclude isotopes within 4 Da; and the number of candidate ions to monitor per cycle: 10. QC samples were prepared by pooling 10 μl of each sample and were analysed approximately once every five injections to monitor the stability and repeatability of the data produced by the instrument.

The raw ultra-high-performance liquid chromatography-quadrupole time-of-flight tandem mass spectrometry (UPLC-Q-TOF/MS) data were converted to mzXML files using ProteoWizardMSconventer tool and then processed using XCMS online software.^8^ Metabolite structure identification used a method of accurate mass matching and secondary spectral matched against in-house tandem MS spectral library (Shanghai Applied Protein Technology, Ltd, Shanghai, China). The parameters in XCMS were set as follows: centwave settings for feature detection: Δm/z= 25 ppm, peakwidth= c (10, 60); obiwarp settings for retention time correction: profStep= 1; and parameters for chromatogram alignment: minfrac= 0.5, bw= 5 and mzwid= 0.025. After being normalised and integrated by using support vector regression, the processed data were uploaded into MetaboAnalyst software for subsequent analysis (www.metaboanalyst.ca).^9^ PCA and orthogonal partial least square discriminant analysis (OPLS-DA) were performed for both positive and negative modes after log transformation and pareto scaling. The variable importance in the projection (VIP) value of each variable in the OPLS-DA model was calculated to indicate its contribution to the classification. The online Kyoto Encyclopedia of Genes and Genomes (KEGG) database (http://www.genome.jp/kegg/, updated: September 14, 2016) was used for the identification of metabolic pathways.^10^

*Proteomics profiling of human serum samples*

Serum pools were depleted of most abundant proteins using an Agilent Human-14 Multiple Affinity Removal System Column (Agilent Technologies). The 10 kDa ultrafiltration tube (Sartorius, Germany) was used for desalination and concentration of low-abundance components. One volume of SDT buffer was added, boiled for 15 min and centrifuged at 14000 *g* for 20 min. The supernatant was quantified with a BCA Protein Assay Kit (Bio-Rad, USA). The sample was stored at -80℃.

For each sample, 20 µg of proteins were mixed with 5×loading buffer and boiled for 5 min. The proteins were then separated on 12.5% SDS-PAGE gel (constant current 14 mA, 90 min) and protein bands were visualized by Coomassie Blue R-250 staining for quality control.

Then 200 µg of proteins for each sample were incorporated into 30 µl SDT buffer (4% SDS, 100 mM dithiothreitol (DTT), 150 mM Tris-HCl pH 8.0). The detergent, DTT, and other low-molecular-weight components were removed using UA buffer (8 M Urea, 150 mM Tris-HCl pH 8.0) by repeated ultrafiltration. 100 µl iodoacetamide (100 mM IAA in UA buffer) was added to block reduced cysteine residues and the samples were incubated for 30 min in darkness. The filters were washed with 100 µl UA buffer three times and then 100 µl 100 mM TEAB buffer twice. Finally, the protein suspensions were digested with 4 µg trypsin (Promega, Wisconsin, USA) in 40 µl TEAB buffer overnight at 37℃, and the resulting peptides were collected as a filtrate. The peptide content was estimated by ultraviolet light (280 nm) using an extinction coefficient of 1.1 of 0.1% (g/l) solution that was calculated based on the frequency of tryptophan and tyrosine in vertebrate proteins.

About 100 μg peptide mixture of each sample was labeled using TMT reagent (Thermo Fisher Scientific Inc., MA, USA) according to the manufacturer's instructions. Pierce high pH reversed-phase fractionation kit (Thermo Fisher Scientific) was used to fractionate TMT-labeled digest samples into 12 fractions by an increasing acetonitrile step-gradient elution according to instructions.

Each fraction was loaded onto a reverse phase trap column connected to the C18-reversed phase analytical column in buffer A (0.1% Formic acid) and separated with a linear gradient of buffer B (84% acetonitrile and 0.1% Formic acid) at a flow rate of 300 nl/min controlled by IntelliFlow technology (Thermo Fisher Scientific) for nano LC-MS/MS analysis*.*

LC-MS/MS analysis was performed on a Q-Exactive mass spectrometer (Thermo Fisher Scientific) that was coupled to Easy nLC for 60 min in Shanghai Applied Protein Technology. The mass spectrometer was operated in positive ion mode. Mass spectrometric data were acquired using a data-dependent top 10 method dynamically by choosing the most abundant precursor ions from the survey scan (300-1,800 m/z) for HCD fragmentation. Automatic gain control (AGC) target was set to 3E6 and maximum inject time was set to 10 min. Dynamic exclusion duration was 40 s. Survey scans were acquired at a resolution of 70,000 at 200 m/z and resolution for HCD spectra was set to 35,000 at 200 m/z, and isolation width was 2 m/z. Normalized collision energy was 30eV and the underfill ratio, which specifies the minimum percentage of the target value likely to be reached at maximum fill time, was defined as 0.1%. The instrument was run with the peptide recognition mode enabled.

Tandem mass spectrometry (MS/MS) spectra was searched using MASCOT engine (Matrix Science, London, UK; version 2.2) embedded into Proteome Discoverer 1.4.Gene ontology (GO) enrichment on one ontologies (biological process) and KEGG pathway enrichment analysis were performed based on the Fisher's exact test, considering the whole quantified protein annotations as a background dataset.

*Animal experiments*

C57BL/6J mice (beginning weight 20-25 g; Vital River, Beijing, China) were housed in cages with free access to food and water under conditions of constant temperature (24± 2℃) and humidity (55± 5%), on a 12-h light to dark cycle (lights on: 07:00–19:00). The drugs were all dissolved in 0.9% NaCl solution. Dextran sulfate sodium (DSS) was bought from MP Biomedicals (Santa Ana, CA). All parameters of disease activity index (DAI) were scored every two days during DSS treatment. When mice were sacrificed, the colon length (starting above the anus to the top of the cecum) was measured. Behavioral tests for DSS-treated mice in the metabolites treatment experiment were performed with DepressionScan (CleverSys, Inc., VA, USA) in August, 2020, while those for healthy mice in the metabolites treatment experiment and DSS-treated mice in the replication experiment were performed with SuperFst, SuperTst, and SuperMaze+ (Shanghai XinRuan Information Technology Co., Ltd., Shanghai, China) in June, 2021. The videos of behavioral tests were analysed by ANY-Maze software.

*Behavioral tests*

Tail Suspension Test (TST)

Each mouse was individually suspended on a tail suspension monitor 2 cm from the tail tip in a suspended state, and its head was more than 10 cm from the floor of the equipment. After 2 min of adaptation, the immobility time was recorded for 4 min using a video tracking system.

Forced Swimming Test (FST)

Each mouse was individually placed in an organic glass tank (height: 40 cm, diameter: 30 cm) filled with water (depth of 20 cm, water temperature 23±2℃). Each mouse was forced to swim in the tank for 6 min with the first 2 min as adaptation, and the immobility duration in the last 4 min was recorded using a video tracking system.

Open Field Test (OFT)

Each mouse was individually and gently placed in a chamber (50 cm× 50 cm× 30 cm) for 1 min of adaptation and then test for 5 min. Its movement pattern was recorded using a video tracking system. The total moving distance and central residence time were measured.

*Data analysis*

Welch's t-test was applied for the KEGG enrichment analysis of 16S rRNA V3-V4 sequencing data. Genus and species associations in each group were estimated using SparCC on the selected genus and species, which significantly differed between patients with UC and depression/anxiety and patients with UC and non-depression/non-anxiety.^11^ Significant co-occurring and co-excluding interactions (SparCC correlation scores ⍴< -0.2 or > 0.2 with *p*< 0.05) were visualised and analysed using *igraph* package. Multi-omics phenotype matrix correlation network was constructed using the modified RV correlation matrix coefficient. Each phenotypic table corresponds to a node, and the edges represent relationships between tables, that is, the percentage of shared similarity. The percentage of shared similarity is derived from the RV^2^ matrix correlation coefficient, corresponding to the proportion of explained variance shared by the two tables. Gut microbiota table includes all the 296 OTUs. Serum metabolome table includes all the 299 metabolites discovered by both positive and negative modes. Lifestyle table includes habits about drinking, smoking, diet, treatment with 5-aminosalicylic acid, enema, pro-biotics and anti-biotics. General information table includes age, gender, BMI, and education level. Psychological state table includes PHQ-9 and GAD-7 scores, which represent depression and anxiety levels, respectively. UC phenotype table includes information about UC disease, namely, history, Mayo score, Mayo endoscopic score, Montreal classification, and Bristol score. Laboratory examination table includes values of Calprotectin, erythrocyte sedimentation Rate, C-reactive protein, hemoglobin, and total protein.

**Supplementary Results**

13% of patients with UC at the Phase 1 had thoughts of suicide. About one third of patients with MDD took antidepressant/anxiolytic drugs in the past three months.

Fecal microbial community richness and diversity was indicated by alpha-diversity. Alpha-diversity is represented by two indices, namely, Shannon index and PD whole tree diversity. As compared to the UCNA group, the UCA group had significantly reduced alpha-diversity at Phase 1 (Shannon index: *p*= 0.038; PD whole tree diversity: *p*= 0.0034), and lower but not significant alpha-diversity at Phase 2 (*p*> 0.05). UCD group had lower but not significant alpha-diversity comparing to UCND group at Phase 1 (Shannon index: *p*= 0.067; PD whole tree diversity: *p*= 0.066).

After quality control, 296, 276, and 344 OTUs of patients in Phase 1, Phase 2, and patients without IBD were retained for subsequent analyses.

In order to explore whether there are specific metabolites that bridge the communication between gut microbiota and mental state, non-target serum metabolomics was performed. After quality control, 5405 and 4352 peaks were identified in positive ion mode (ES+) and negative ion mode (ES-), respectively, of which 299 compounds can be identified. We additionally explored serum proteome via TMT quantitative proteomics detection method to find proteins associated with UC-related depression/ anxiety. Most metabolites in the network were up-regulated in patients with UC and depression/ anxiety, while most proteins in the network were down-regulated.

In non-IBD population, patients with major depression disorder (the MDD group) harbored more *Sellimonas* (Student's t-test, *p*< 0.05), less *Erysipelotrichaceae UCG-003* (Student's t-test, *p*< 0.05), in consistent with the results in UC population (Supplemental Table S2).

The crosstalk among different omics data was quantified by estimating the proportion of shared variation using RV2 coefficients analysis with Phase 2 data. Gut microbiota shared the largest (18%) variation with serum metabolome (*p*= 0.13), and 17% with participants' lifestyle (*p*= 0.018, Supplementary Figure S6). UC phenotype shared 13% similarity with laboratory examination results (*p*= 0.004); serum metabolome shared 10% similarity with subject's general information (*p*= 0.027), suggesting a mild similarity between multi-omics data.

We additionally tried to characterize gut microbiota in these mice to find similarity and difference with the gut microbiota in human subjects. *Verrucomicrobia* and its subordinate bacteria (including *Akkermansia*), *Enterococcaceae* and its subordinate *Enterococcus*, and *Streptococcus* were increased when mice were induced with colitis accompanied by depression and anxiety, in accordance with enrichment of these microbes in patients with UC with depression/ anxiety (by meta analysis). Among these microbes, *Verrucomicrobia* and its subordinate bacteria (including *Akkermansia*) could be reduced by application of the four metabolites (Supplementary Figure S12). On the other hand, *Dorea* and *Subdoligranulum* were reduced when mice were induced with colitis accompanied by depression and anxiety, and this trend could be reversed by application of the four metabolites. These two bacteria were also decreased in patients with UC and depression/ anxiety (by either Student's t-test or general linear model).

Compared with DSS-treated mice with little or none depressive-like behavior, DSS-treated mice with more depressive-like behavior harbored less L-pipecolic acid in the serum and brain, and less hydroxyphenyllactic acid in the serum. They had less L-gulonic gamma-lactone, but more glycocholic acid, 1-stearoyl-rac-glycerol, 1-stearoyl-2-arachidonoyl-sn-glycerol, alpha-tocopherol, 3-indolepropionic acid, D-quinovose, and pentadecanoic acid in the serum; and they had more 1-stearoyl-rac-glycerol, 1-stearoyl-2-hydroxy-sn-glycero-3-phosphocholine, 1-stearoyl-2-arachidonoyl-sn-glycerol, 1-O-(cis-9-octadecenyl)-2-O-acetyl-sn-glycero-3-phosphocholine, and 1-stearoyl-2-oleoyl-sn-glycerol-3-phosphocholine in the brain. The trend of these above metabolites is consistent with that in the human serum metabolome (*p*> 0.05, Student's t-test). Levels of 20-hydroxyarachidonic acid, acetylcarnitine, cytosine, erucamide, and guanosine in the serum and brain were positively correlated (*p*< 0.05, Spearman's rank correlation test).

Compared with the control mice, DSS-treated mice showed increased levels of IL-1β, IL-6, TNF-α, and LBP in the colon (*p*_IL-1β_= 1.0× 10^-8^, *p*_IL-6_= 0.00098, *p*_TNF-α_= 0.00026, *p*_LBP_= 1.1×10^-6^, Figure 5D), increased levels of IL-1β and IL-6 in the serum (*p*_IL-1β_= 0.001, *p*_IL-6_= 1.2×10^-5^, Supplementary Figure S13), and increased levels of IL-6, TNF-α and LBP in the brain (*p*_IL-6_= 0.00088, *p*_TNF-α_= 0.0041, *p*_LBP_= 0.32, Supplementary Figure S13). Compared with the mice without depressive-like behavior, t the mice with depressive-like behavior showed increased IL-6, TNF-α and LPS in the colon, elevated IL-1β, IL-6 and TNF-α in the serum, and elevated LPS and LBP in the brain (*p*> 0.05, Student's t-test, Supplementary Figure S11).

2'-deoxy-D-ribose, L-pipecolic acid, and hydroxyphenyllactic acid were able to reduce the level of IL-6 in the colon of mice with depressive-like behavior (*p*_2'-deoxy-D-ribose_= 0.09, *p*_L-pipecolic acid_= 0.10). 2'-deoxy-D-ribose (*p*_IL-1β_= 1.2×10^-12^, *p*_IL-6_= 4.0×10^-9^, *p*_TNF-α_= 5.2×10^-7^) and L-pipecolic acid (*p*_IL-1β_= 6.1×10^-10^, *p*_IL-6_= 1.5×10^-6^, *p*_TNF-α_= 2.1×10^-6^) significantly reduced serum IL-1β, IL-6 and TNF-α levels in mice with colitis, while hydroxyphenyllactic acid significantly reduced serum IL-1β and IL-6 levels in mice with colitis (*p*_IL-1β_= 7.2×10^-5^, *p*_IL-6_= 3.1×10^-3^) (Figure 5D, Supplementary Figure S13). Besides, 2'-deoxy-D-ribose could also reduce the elevated IL-1β (*p*= 3.1×10^-6^), IL-6 (*p*= 6.6×10^-5^) and TNF-α (*p*= 2.3×10^-7^) levels in the hippocampus of the DSS-treated mice's brain (Figure 5D, Supplementary Figure S13). Similar pattern was observed for the L-pipecolic acid treated group (*p*_IL-6_= 2.7×10^-7^, *p*_TNF-α_= 4.8×10^-7^). Administration of 2'-deoxy-D-ribose and L-pipecolic acid reduced serum levels of IL-1β, IL-6 and TNF-α in non-DSS healthy mice. However, treatment with these four metabolites increased pro-inflammatory cytokines levels in the brain(*p*< 0.1, Student's t-test). 4-hydroxybenzoate increased the levels of pro-inflammatory cytokines in the serum and brain of mice (*p*< 0.05, Student's t-test, Figure 5D, Supplementary Figure S13).

We further investigated changes in selected neurotransmitters in the blood and hippocampus, a publically reported brain region involved in depression, due to colitis and treatment of these beneficial metabolites. As dopamine was significantly elevated in the blood when patients with UC exhibit depression symptoms, we then tried to replicate this result in the mice model and to investigate whether administration of these metabolites could alleviate dopamine levels. Compared with the control group, the dopamine level in the serum and hippocampus of mice treated with DSS increased significantly (*p*_blood_= 0.0024, *p*_brain_= 0.0031). Administration of 2'-deoxy-D-ribose and L-pipecolic acid significantly decreased dopamine level in DSS-treated mice, consistent with results in the human metabolomics (2'-deoxy-D-ribose: *p*_blood_= 3.6×10^-10^, *p*_brain_= 3.0×10^-10^; L-pipecolic acid: *p*_blood_= 1.8×10^-6^, *p*_brain_= 0.0012). 2'-deoxy-D-ribose and L-pipecolic acid decreased the dopamine level in the serum in healthy mice, but increased the dopamine level in the brain (*p*< 0.01, Student's t-test) (Supplementary Figure S13A).

In addition, we found that TMEM119, a marker for stationary microglia, decreased in the brain after DSS treatment (*p*= 0.00029, Student's t-test, Supplementary Figure S11B). Compared with colitis mice without depressive-like behavior, level of TMEM119 mRNA in the brain of mice with depressive-like behavior decreased (*p*= 0.13, Student's t-test, Supplementary Figure S11C), and 2'-deoxy-D-ribose, L-pipecolic acid and hydroxyphenyllactic acid could increase its expression (*p*_2'-deoxy-D-ribose_= 0.105, *p*_L-pipecolic acid_= 0.015, *p*_hydroxyphenyllactic acid_= 0.29, Supplementary Figure S11B).

In the mice treated with DSS to induce colitis and then rescued with selected metabolites, we further quantified the immune-related proteins which were closely associated with the selected metabolites. One of the most closely associated protein is IGHV3, as its several family members were significantly associated with UCD/UCA phenotype, namely, IGHV3-74, IGHV3-9, IGHV3-49, IGHV3-7, and IGHV3-30. We found that serum IGHV3 protein, which was significantly reduced when mice were induced of colitis by DSS (*p*= 0.0034), was significantly increased when mice were treated with 2'-deoxy-D-ribose and hydroxyphenyllactic acid (*p*_2'-deoxy-D-ribose_= 3.1×10^-12^; *p*_hydroxyphenyllactic acid_= 2.0×10^-4^, Supplementary Figure S13). IGKV3 protein was lower in DSS-treated mice (*p*= 0.28), and significantly increased in mice treated with L-pipecolic acid and hydroxyphenyllactic acid (*p*_L-pipecolic acid_= 3.5×10^-9^; *p*_hydroxyphenyllactic acid_= 6.4×10^-12^, Supplementary Figure S13). These results coincided with the clinical results that 2'-deoxy-D-ribose was significantly correlated with a set of IGHV3 proteins (IGHV3-9, IGHV3-74), while L-pipecolic acid was significantly correlated with IGKV3D-11, a subtype of IGKV3 protein. It further validates the association between metabolites and proteins in patients with UC and depression.

**Supplementary Discussion**

*Lachnospiraceae* and *Ruminococcaceae*, accounted for 38%-43% of the identified gut microbiota in patients with active UC and depression/ anxiety, could produce butyric acid, a subtype of short-chain fatty acids (SCFAs), which can affect the brain.^12,13^ Serum metabolomics identified a type of SCFA, valeric acid, demonstrating a reduction in patients with UC and depression/ anxiety with borderline significance. The increase of *Bacteroidaceae* in the patients with depression and anxiety is consistent with a previous study that mice monocolonized with *Bacteroides fragilis,* which could produce GABA, display deficits in serum serotonin.^14,15^ *Ruminococcus* is significantly associated with gut inflammation.^16^ There is few report about the relationship of mood and *Sellimonas*, which needs further research about their functional significance. *Prevotella* is associated with hippocampus's function and structure.^17^ Indole-3-propionic acid has the potential to treat Alzheimer's disease, but its relationship with depression remains still unclear, which is worthy further research.^18^ These bacteria, metabolites, and proteins may interact with each other through synergistic or antagonistic interactions.

**
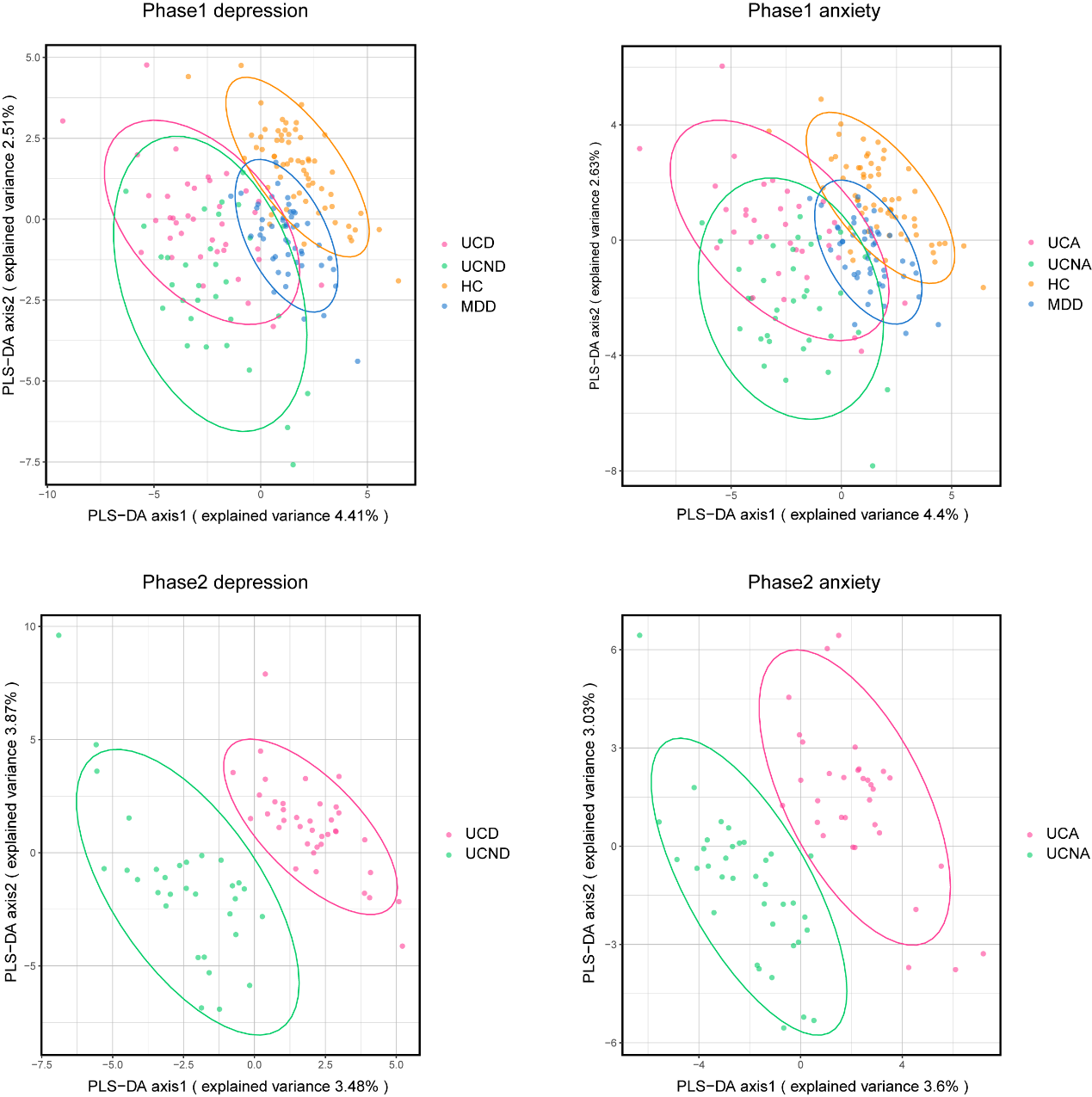
**

**Figure S1.** PLS-DA score plots based on the microbiome profiles of depression and anxiety phenotype in the two phases.


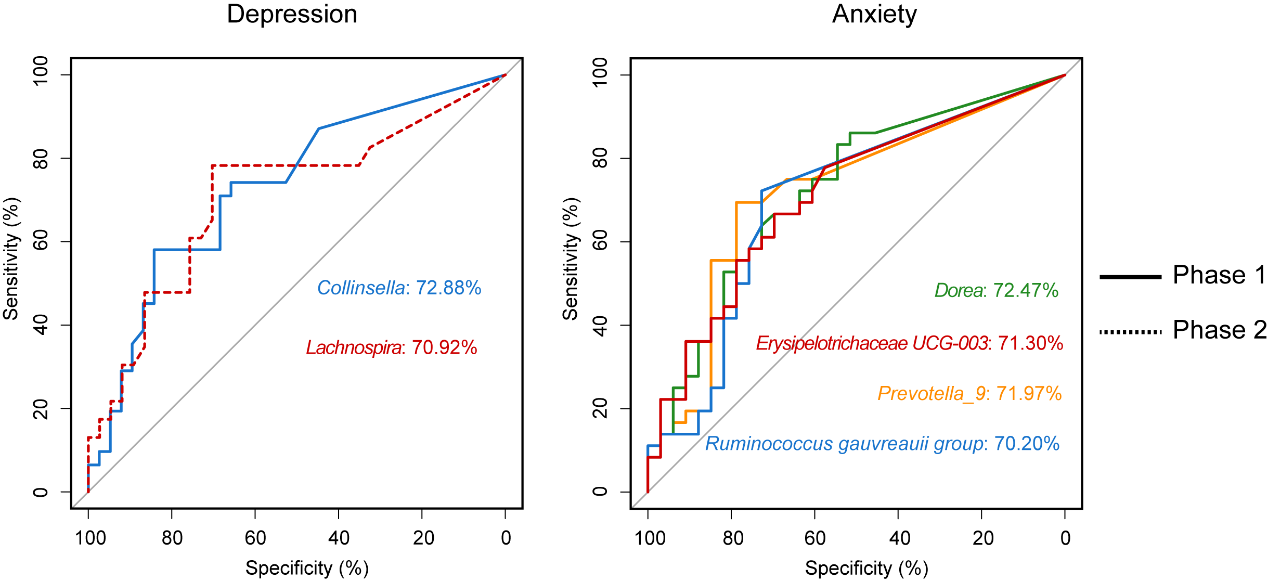


**Figure S2.** Diagnostic ability of some bacteriaon depression and anxiety levels (indicated by PHQ-9 and GAD-7 scores) in UC patients at either Phase 1 (solid lines) or Phase 2 (dotted lines) based on ROC. Only those with AUC> 0.7 were displayed.


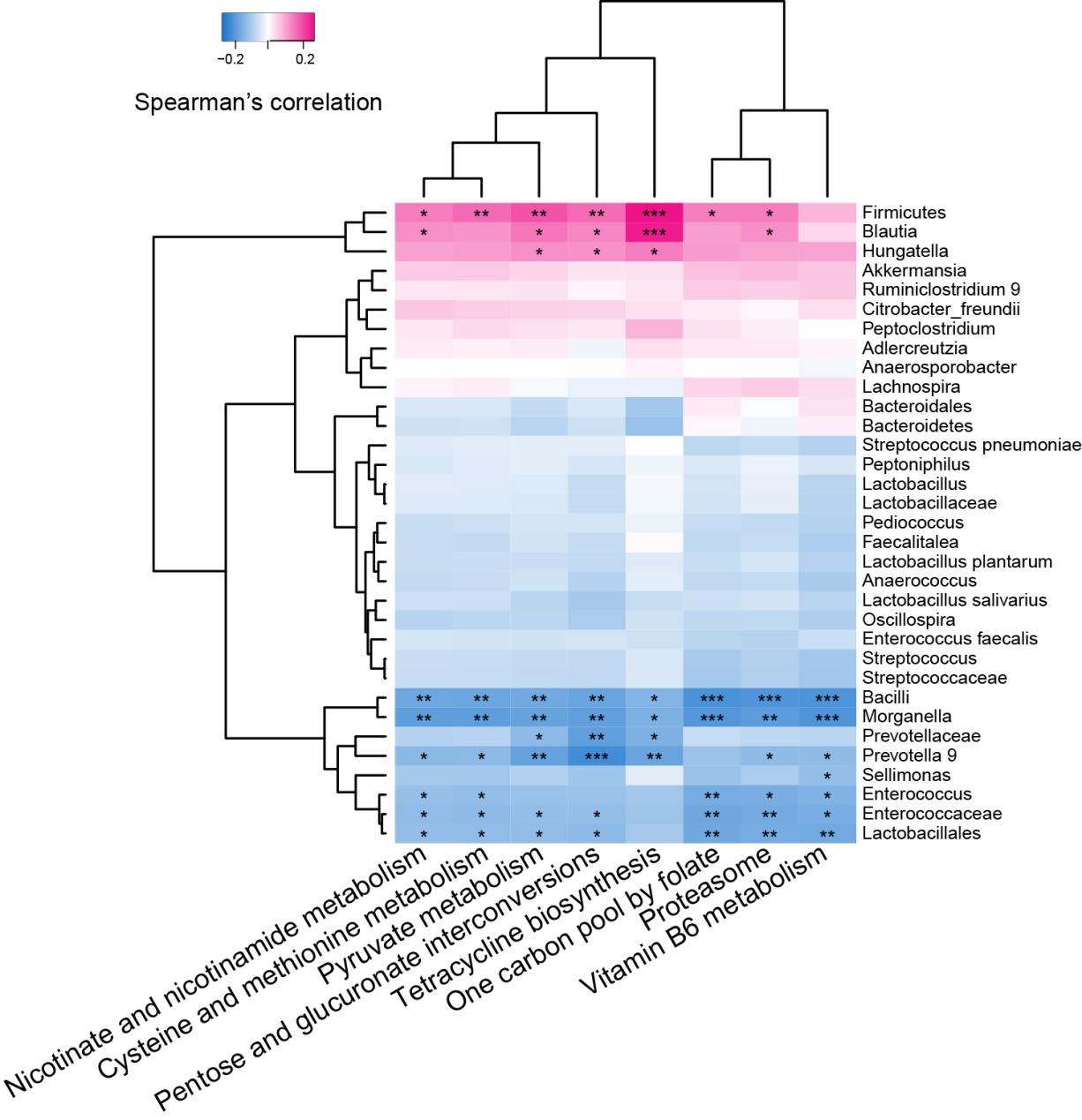


**Figure S3.** Spearman's correlation between KEGG pathways and microbial community richness and diversity. Significantly enriched KEGG pathways were derived from Welch’s t-test of PICRUSt-HUMAnN2. Significance levels were indicated by asterisks as **p*< 0.05, ***p*< 0.01, and ****p*< 0.001.


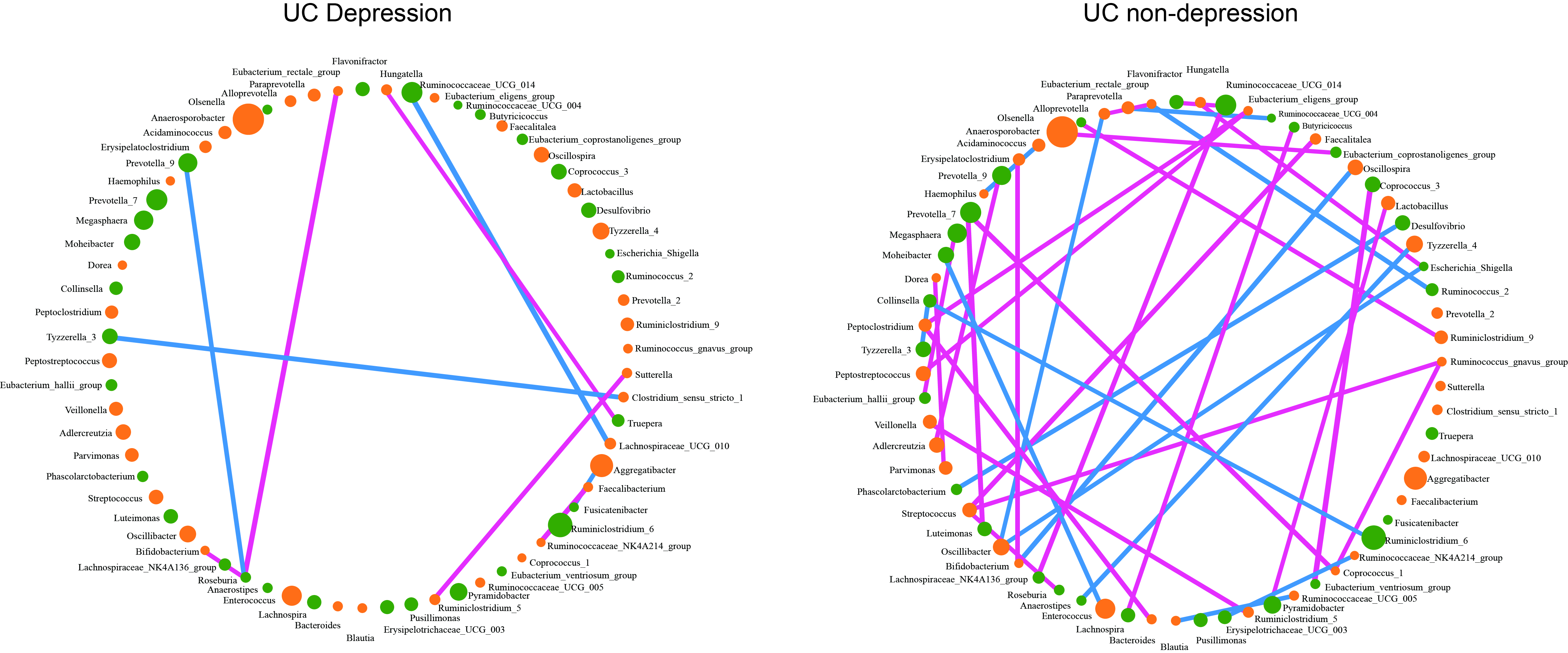


**Figure S4.** Cluster relationships between taxa in UCND (right) and UCD (left) in the combined cohort of the two phases. Only SparCC correlation with |⍴|> 0.2 and *p*< 0.05 is shown. The edge width corresponds to SparCC correlation coefficients. The node size reflects fold change of UCD relative to UCND.

**
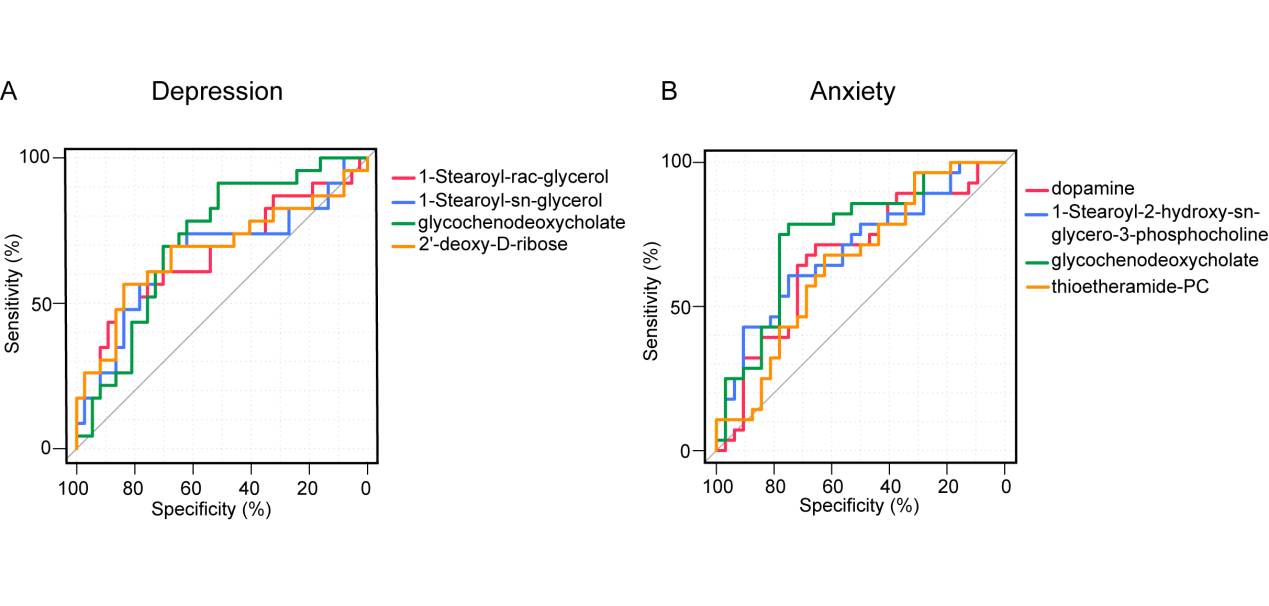
**

**Figure S5.** Diagnostic ability of some metabolites on depression and anxiety levels (indicated by PHQ-9 and GAD-7 scores) in UC patients based on ROC. Only those with AUC> 0.6 were displayed.

**
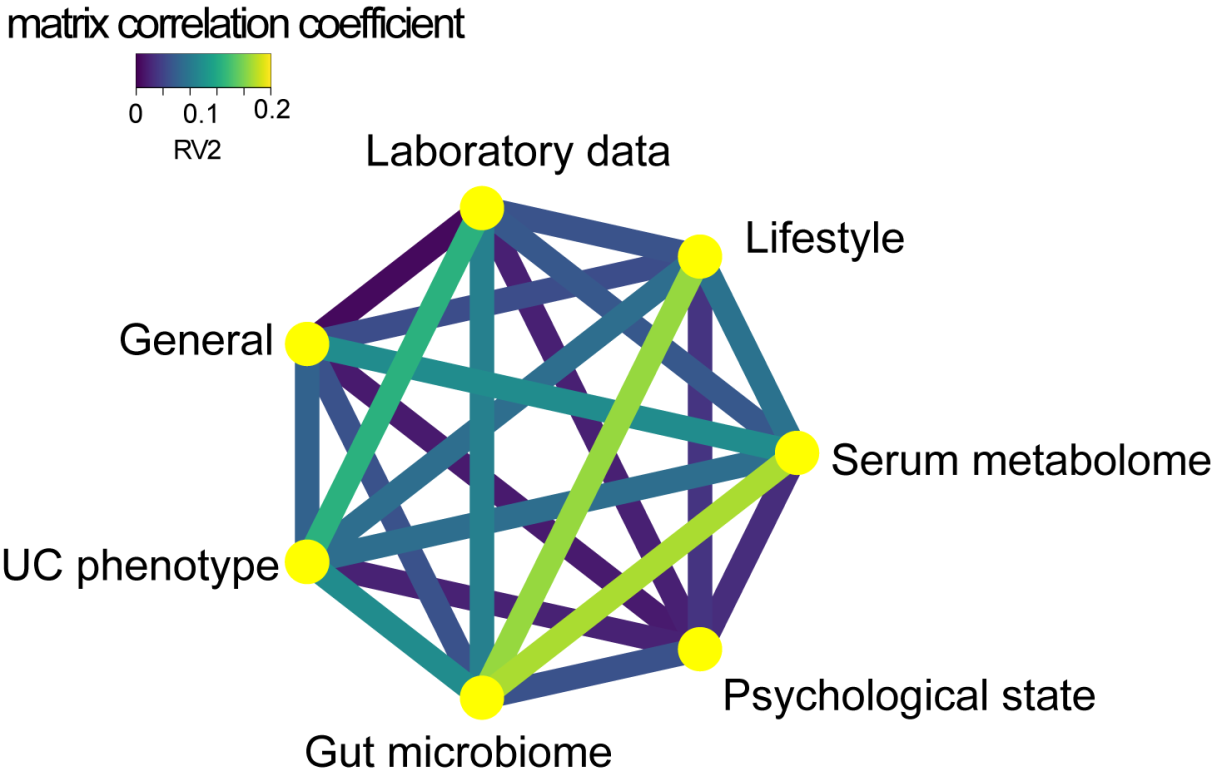
**

**Figure S6.** Multi-omics phenotype matrix correlation network computed for the patients with matching multiple omics profiles (n= 60) using the modified RV correlation matrix coefficient. Each data table corresponds to a node, and the edges represent the relationships between tables, that is, the percentage of shared similarity, derived from the RV^2^ matrix correlation coefficient corresponding to the proportion of variance shared by the two tables. The color of each edge corresponds to the magnitude of RV2 coefficient (ranges -1 to 1) between the two tables. General means general information of subjects such as age, gender, and BMI.

**
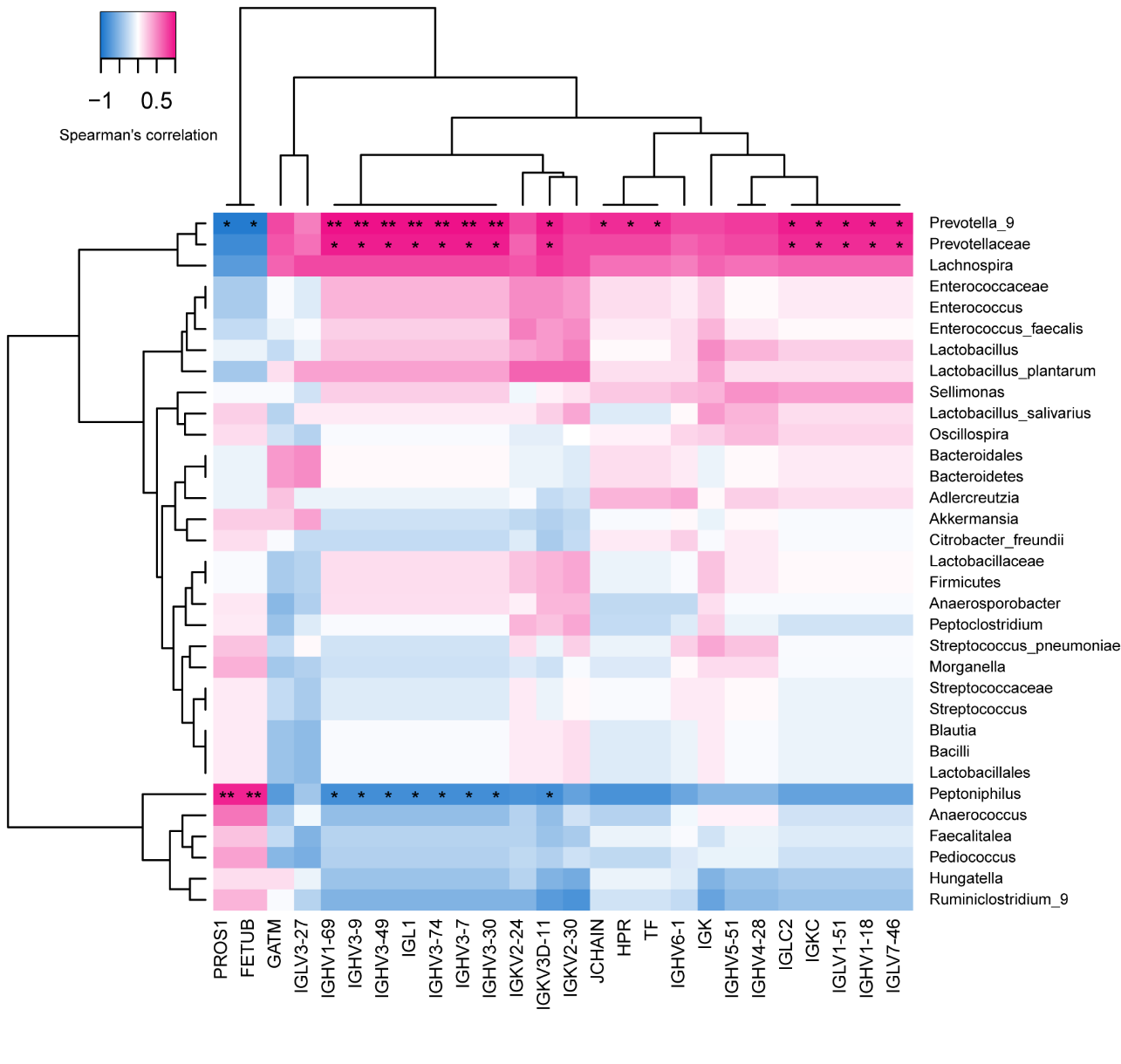
**

**Figure S7.** Spearman’s correlation between phenotype associated bacteria and proteins. Only proteins with significant statistical difference (FDR< 0.05, Student’s t-test) between UCD/UCA group and UCND/UCNA group are showed. Significance levels are indicated by asterisks as **p*< 0.05, ***p*< 0.01, and ****p*< 0.001.


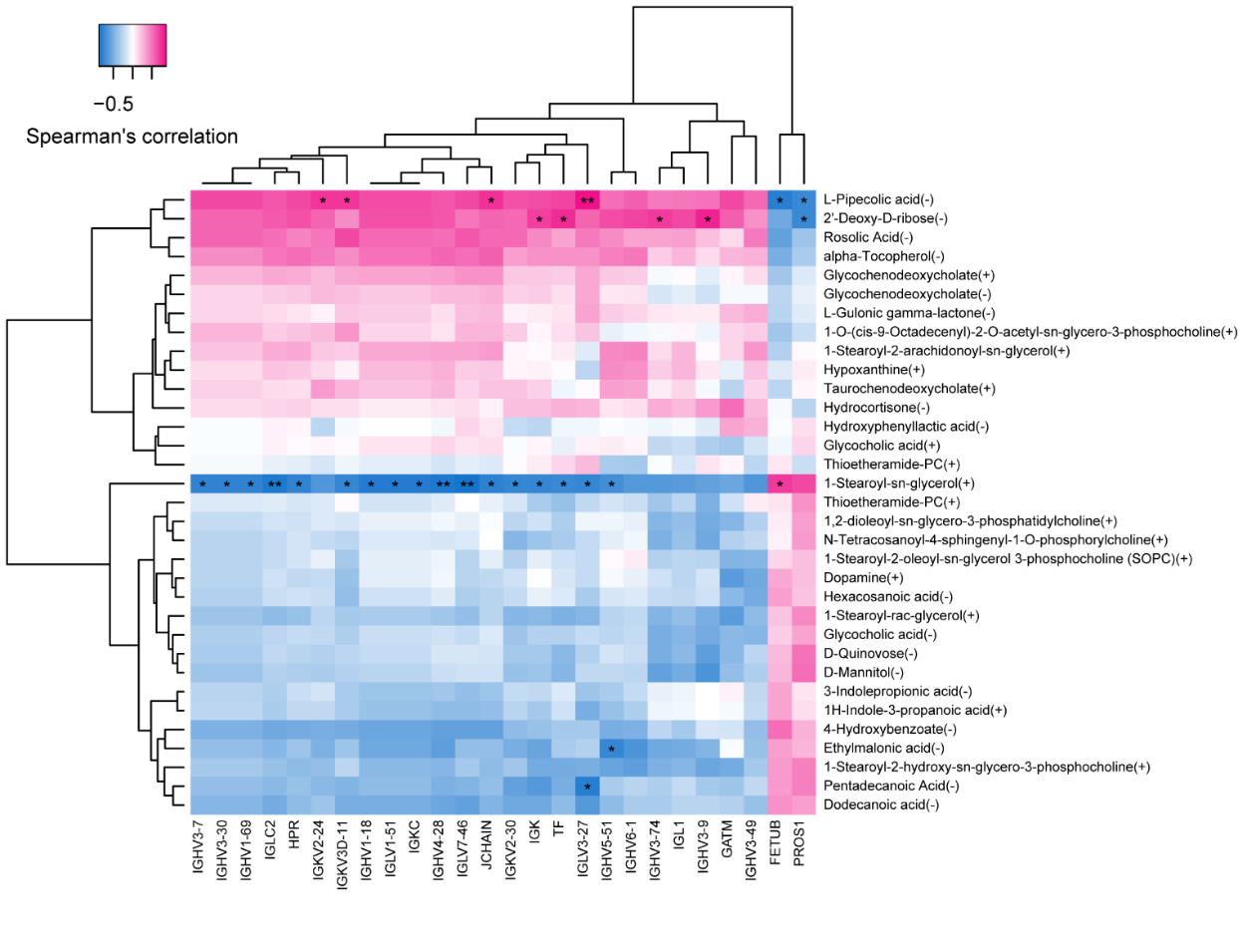


**Figure S8.** Spearman’s correlation between phenotype associated metabolites and proteins. Only proteins with significant statistical difference (FDR< 0.05, Student’s t-test) between UCD/UCA group and UCND/UCNA group are showed. Significance levels were indicated by asterisks as **p*< 0.05, ***p*< 0.01, and ****p*< 0.001.


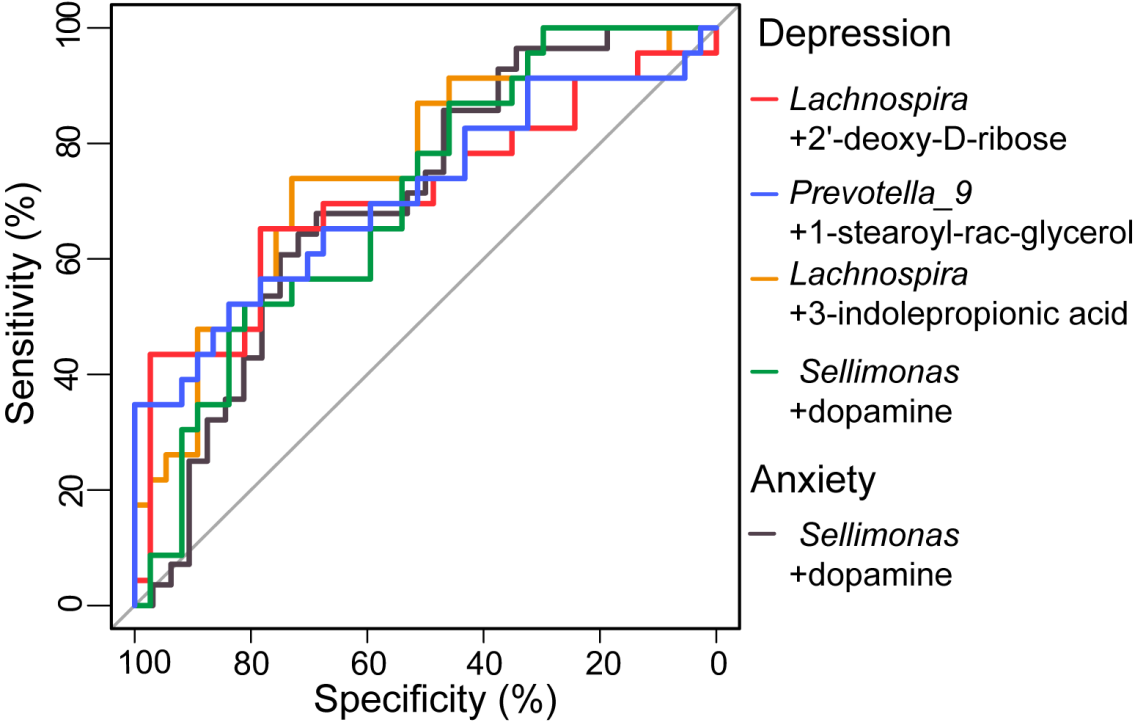


**Figure S9.** Diagnostic ability of some bacteria-metabolite combinations on depression and anxiety in UC patients based on ROC. Only those combination with AUC> 70% are shown.


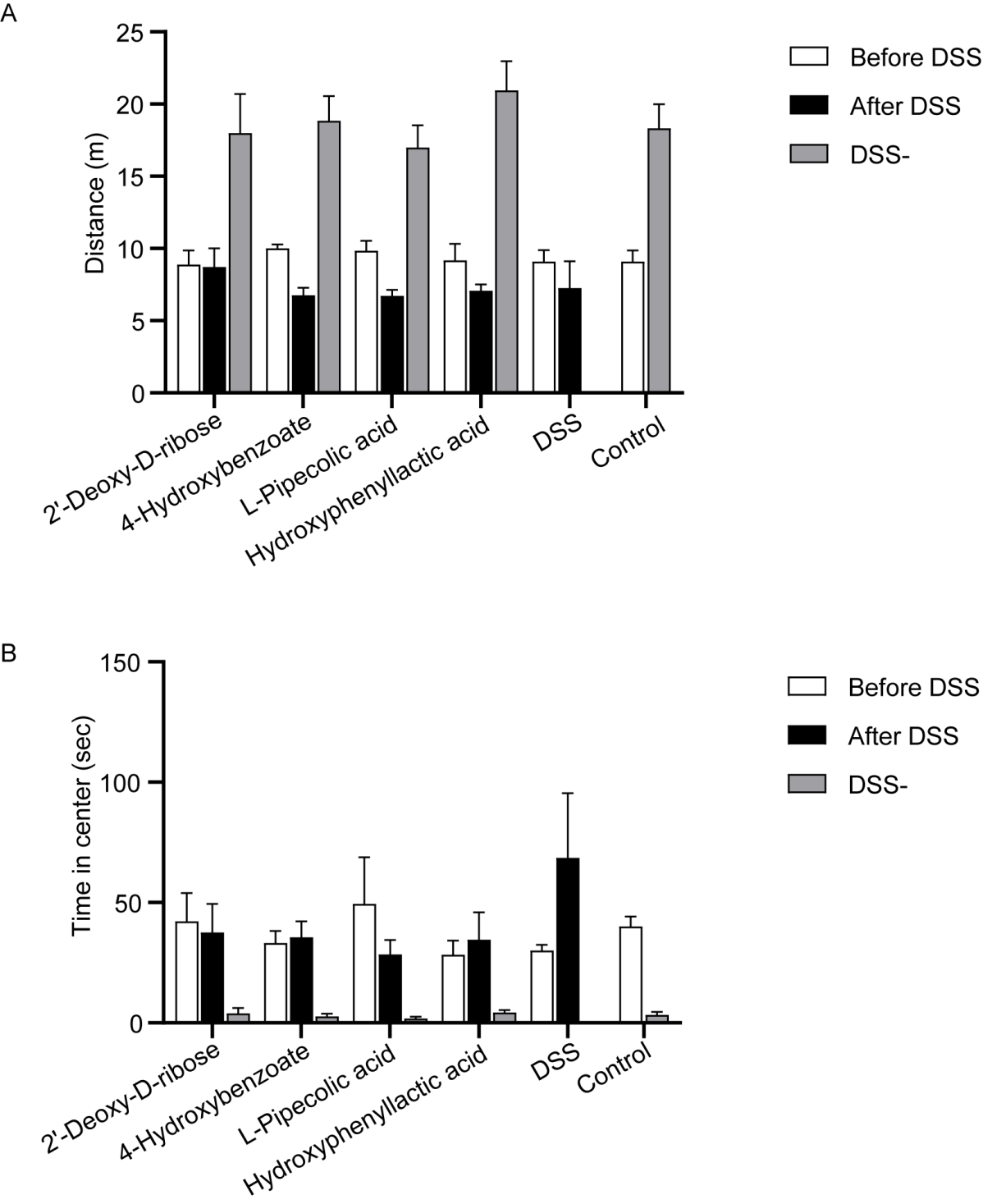


**Figure S10.** The effect of selected metabolites on DSS-treated mice's anxious-like behavior, assessed by open field test, in terms of (A) the total distance moved in meters (m) and (B) the time spent in the central area in seconds (sec). Bars represent means ± standard error of mean. Before /After DSS, before /after dextran sulfate sodium treatment. DSS-, without DSS treatment.


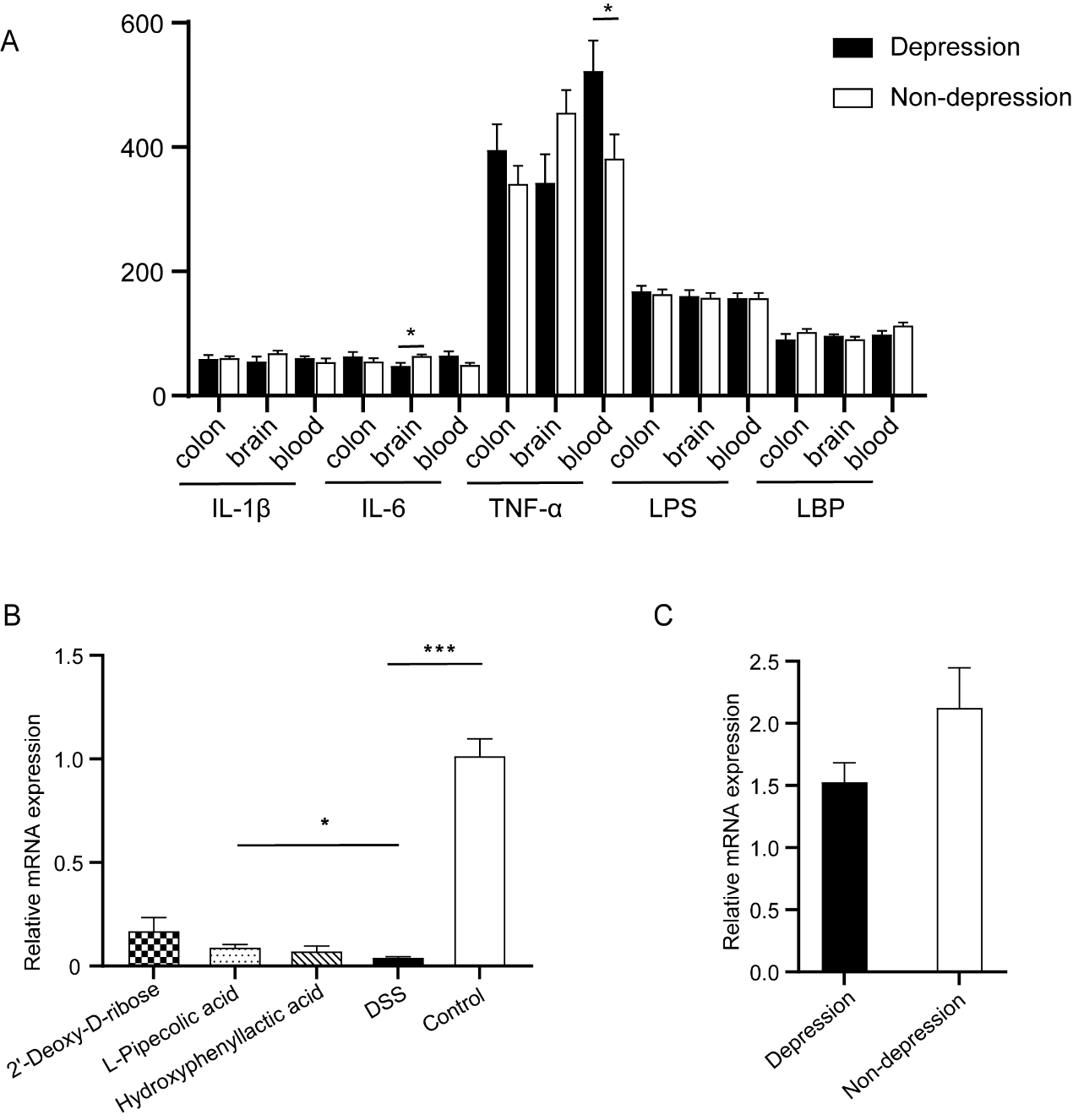


**Figure S11.** Changes of inflammation related proteins in response to DSS and metabolites treatments. (A) Differences of inflammation-related factors in DSS-treated mice with (Depression) and without (Non-depression) depressive-like behavior in the replication experiment. Expression of TMEM119 (a marker for stationary microglia) in the brain of mice in the metabolite treatment experiment (B) and the replication experiment (C). Bars represent means ± standard error of mean. Significance levels are indicated by asterisks as **p*< 0.05, ***p*< 0.01, and ****p*< 0.001.

**
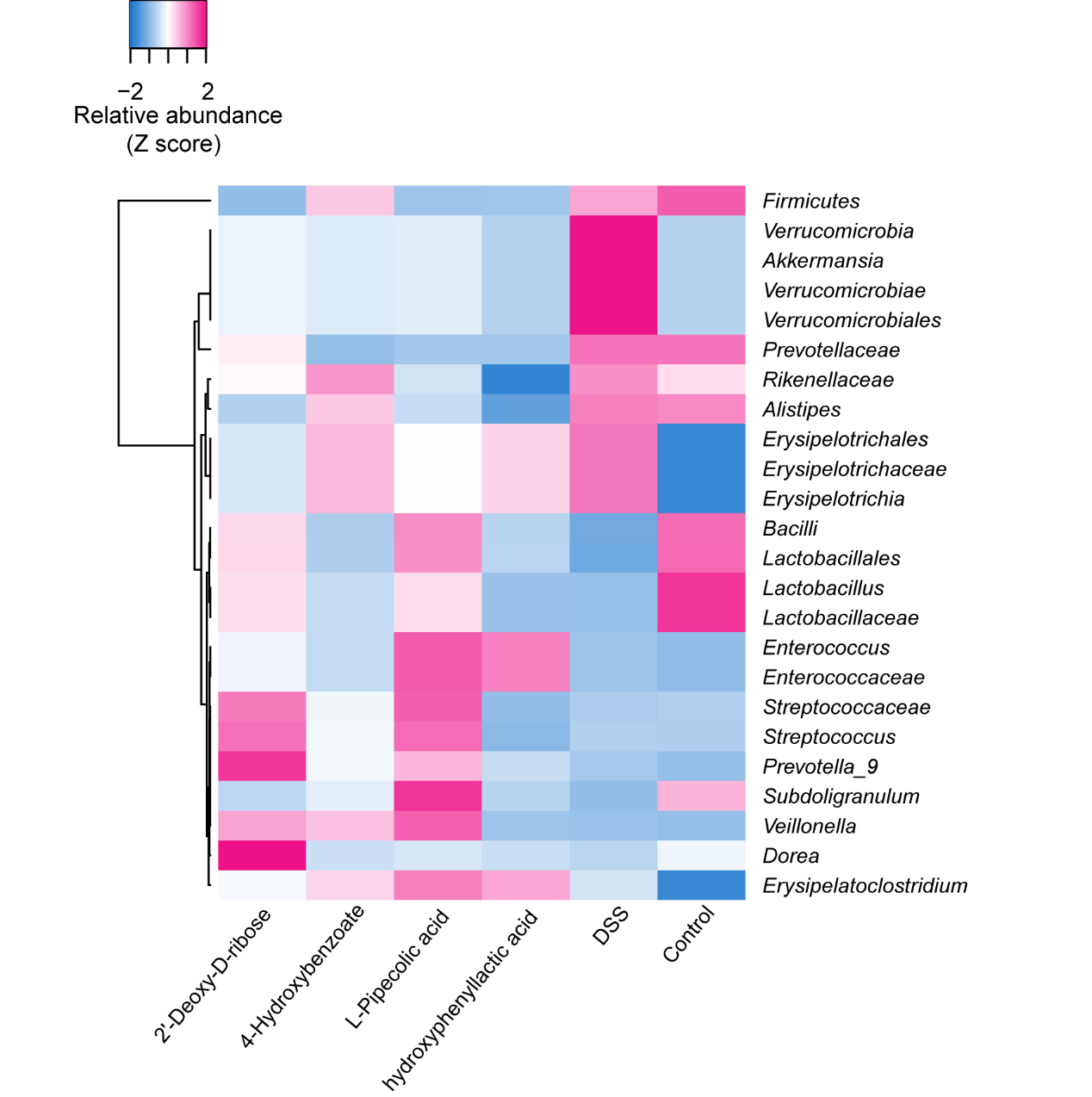
**

**Figure S12.** Relative abundances of the microbes in mice. These microbes were revealed to be significantly associated with UC depression/ anxiety in human subjects either by Student's t-test or by general linear model, and were also identified in mice cecal samples. Abundance is standardized across samples for each microbe.


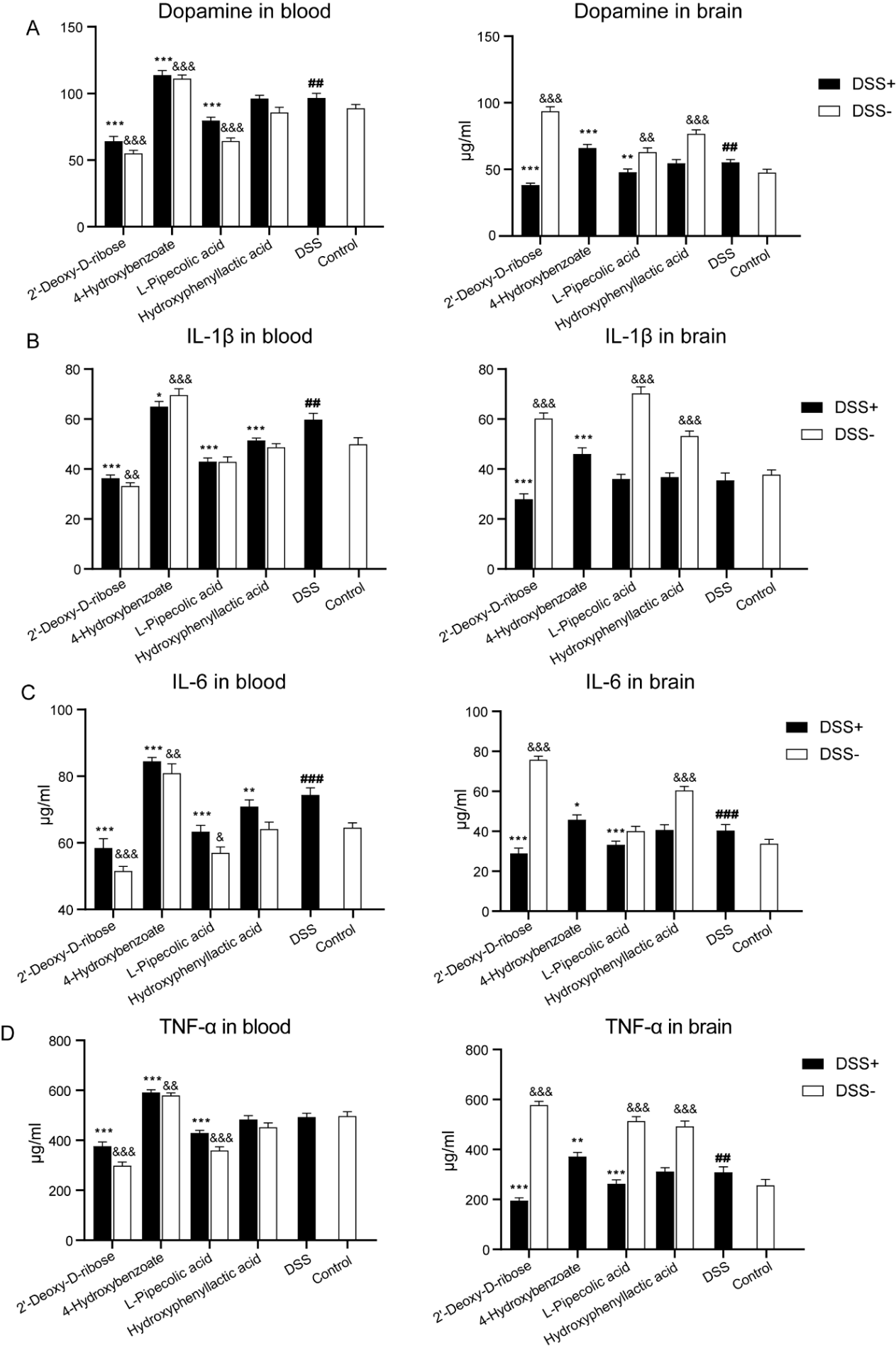


**Figure S13.** Quantification of dopamine and pro-inflammatory cytokines in the serum and brain of DSS-treated (DSS+) and healthy mice (DSS-) under intervention of metabolites. Bars represent means ± standard error of mean. *Significant differences between DSS model and metabolite-treated mice in DSS+ groups; #Significant differences between DSS model and control in DSS+ groups; &Significant differences between DSS model and control in DSS- groups.


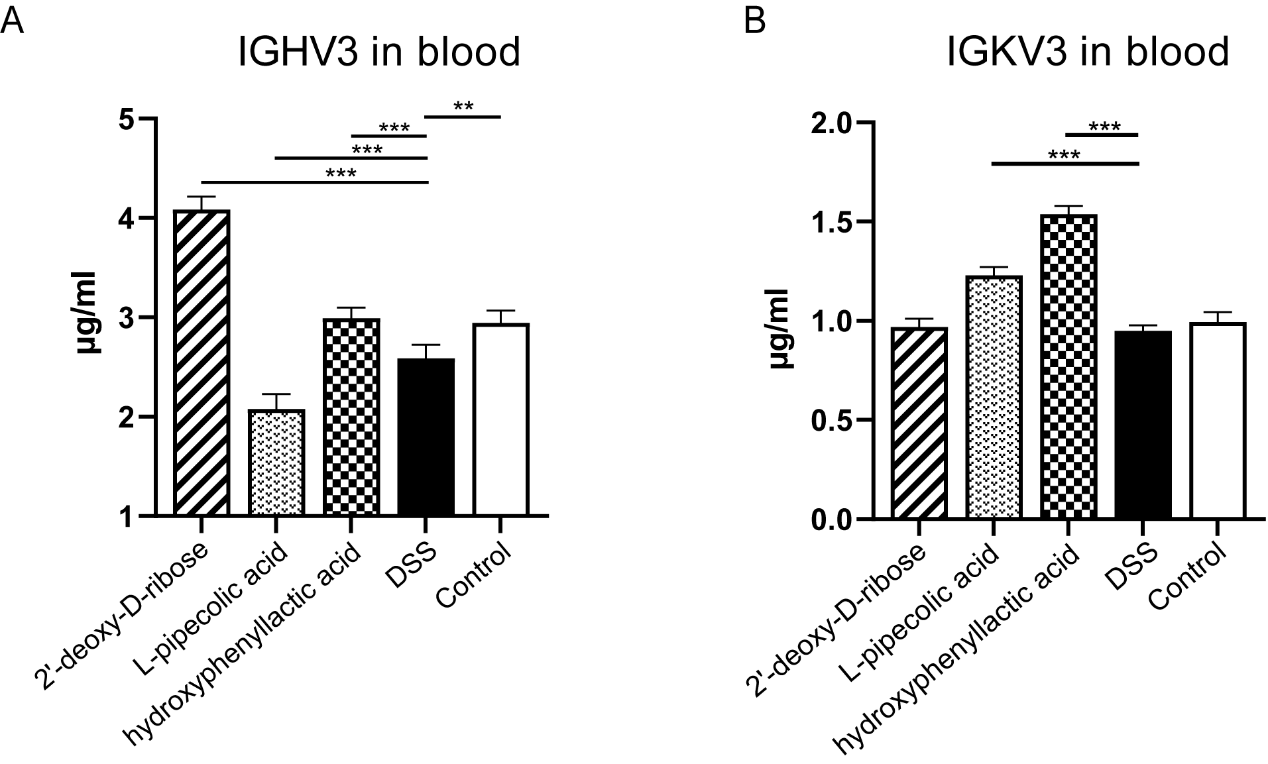


**Figure S14.** Quantification of IGHV3 and IGKV3 in the serum of DSS-treated mice under intervention of different metabolites. Bars represent means ± standard error of mean. Significance levels were indicated by asterisks as **p*< 0.05, ***p*< 0.01 and ****p*< 0.001.

**References**

1. Kroenke K, Spitzer RL, Williams JB. The PHQ-9: validity of a brief depression severity measure. J Gen Intern Med 2001; 16:606–13.

2. Löwe B, Decker O, Müller S, Brähler E, Schellberg D, Herzog W, Herzberg PY. Validation and standardization of the Generalized Anxiety Disorder Screener (GAD-7) in the general population. Med Care 2008; 46:266–74.

3. Munyaka PM, Eissa N, Bernstein CN, Khafipour E, Ghia J-E. Antepartum Antibiotic Treatment Increases Offspring Susceptibility to Experimental Colitis: A Role of the Gut Microbiota. PLoS One 2015; 10:e0142536.

4. Edgar RC. UPARSE: highly accurate OTU sequences from microbial amplicon reads. Nat Methods 2013; 10:996–8.

5. Cole JR, Wang Q, Cardenas E, Fish J, Chai B, Farris RJ, Kulam-Syed-Mohideen AS, McGarrell DM, Marsh T, Garrity GM, et al. The Ribosomal Database Project: improved alignments and new tools for rRNA analysis. Nucleic Acids Res 2009; 37:D141-5.

6. Wang Y, Sheng H-F, He Y, Wu J-Y, Jiang Y-X, Tam NF-Y, Zhou H-W. Comparison of the levels of bacterial diversity in freshwater, intertidal wetland, and marine sediments by using millions of illumina tags. Appl Environ Microbiol 2012; 78:8264–71.

7. Jiang X-T, Peng X, Deng G-H, Sheng H-F, Wang Y, Zhou H-W, Tam NF-Y. Illumina sequencing of 16S rRNA tag revealed spatial variations of bacterial communities in a mangrove wetland. Microb Ecol 2013; 66:96–104.

8. Smith CA, Want EJ, O’Maille G, Abagyan R, Siuzdak G. XCMS: processing mass spectrometry data for metabolite profiling using nonlinear peak alignment, matching, and identification. Anal Chem 2006; 78:779–87.

9. Chong J, Wishart DS, Xia J. Using MetaboAnalyst 4.0 for Comprehensive and Integrative Metabolomics Data Analysis. Curr Protoc Bioinforma 2019; 68:e86.

10. Kanehisa M, Goto S, Sato Y, Furumichi M, Tanabe M. KEGG for integration and interpretation of large-scale molecular data sets. Nucleic Acids Res 2012; 40:D109-14.

11. Friedman J, Alm EJ. Inferring correlation networks from genomic survey data. PLoS Comput Biol 2012; 8:e1002687.

12. Sowa-Kućma M, Styczeń K, Siwek M, Misztak P, Nowak RJ, Dudek D, Rybakowski JK, Nowak G, Maes M. Lipid Peroxidation and Immune Biomarkers Are Associated with Major Depression and Its Phenotypes, Including Treatment-Resistant Depression and Melancholia. Neurotox Res 2018; 33:448–60.

13. Dalile B, Van Oudenhove L, Vervliet B, Verbeke K. The role of short-chain fatty acids in microbiota-gut-brain communication. Nat Rev Gastroenterol Hepatol 2019; 16:461–78.

14. Strandwitz P, Kim KH, Terekhova D, Liu JK, Sharma A, Levering J, McDonald D, Dietrich D, Ramadhar TR, Lekbua A, et al. GABA-modulating bacteria of the human gut microbiota. Nat Microbiol 2019; 4:396–403.

15. Yano JM, Yu K, Donaldson GP, Shastri GG, Ann P, Ma L, Nagler CR, Ismagilov RF, Mazmanian SK, Hsiao EY. Indigenous bacteria from the gut microbiota regulate host serotonin biosynthesis. Cell 2015; 161:264–76.

16. Schirmer M, Garner A, Vlamakis H, Xavier RJ. Microbial genes and pathways in inflammatory bowel disease. Nat Rev Microbiol 2019; 17:497–511.

17. Labus JS, Hollister EB, Jacobs J, Kirbach K, Oezguen N, Gupta A, Acosta J, Luna RA, Aagaard K, Versalovic J, et al. Differences in gut microbial composition correlate with regional brain volumes in irritable bowel syndrome. Microbiome 2017; 5:49.

18. Abildgaard A, Elfving B, Hokland M, Wegener G, Lund S. The microbial metabolite indole-3-propionic acid improves glucose metabolism in rats, but does not affect behaviour. Arch Physiol Biochem 2018; 124:306–12.
